# CTCF Expression and Dynamic Motif Accessibility Modulates Epithelial–Mesenchymal Gene Expression

**DOI:** 10.3390/cancers14010209

**Published:** 2022-01-01

**Authors:** Kelsey S. Johnson, Shaimaa Hussein, Priyanka Chakraborty, Arvind Muruganantham, Sheridan Mikhail, Giovanny Gonzalez, Shuxuan Song, Mohit Kumar Jolly, Michael J. Toneff, Mary Lauren Benton, Yin C. Lin, Joseph H. Taube

**Affiliations:** 1 Department of Biology, Baylor University, Waco, TX 76706, USA; kelsey_johnson1@baylor.edu (K.S.J.); arvind_muruganantha1@baylor.edu (A.M.); sheridan_mikhail1@baylor.edu (S.M.); giovanny_gonzalez1@alumni.baylor.edu (G.G.); shuxuan_song1@baylor.edu (S.S.); 2 Baylor Institute for Immunology Research, Baylor Scott & White, Dallas, TX 75246, USA; Shaimaa.zaki@gmail.com (S.H.); linchunyin@gmail.com (Y.C.L.); 3 Centre for BioSystems Science and Engineering, Indian Institute of Science, Bangalore 560012, India; priyanka08993@gmail.com (P.C.); mkjolly@iisc.ac.in (M.K.J.); 4 Department of Biology, Widener University, Chester, PA 19013, USA; mtoneff@widener.edu; 5 Department of Computer Science, Baylor University, Waco, TX 76706, USA; marylauren_benton@baylor.edu; 6 Dan L. Duncan Cancer Center, Houston, TX 76706, USA

**Keywords:** EMT, MET, partial EMT, ATAC-seq, chromatin accessibility, CTCF, E-cadherin

## Abstract

**Simple Summary:**

Epithelial–mesenchymal transition (EMT) facilitates cell migration, invasion, and, consequently, metastasis, which ultimately contributes to breast-cancer-related fatalities. In this study, we define the DNA accessibility dynamics that permit EMT and its reversal, MET. We demonstrate the progressive repression of E-cadherin, beginning with the loss of the membrane-bound fraction, and followed by the loss of *CDH1* reporter expression. We identify that EMT is characterized by a global increase in accessible chromatin—nearly doubling the number of accessible regions. Furthermore, we find that regions exhibiting chromatin alterations are enriched in binding motifs for CTCF. Additionally, our data suggest that CTCF repression slows the loss of epithelial gene expression while accelerating the gain of mesenchymal gene expression, facilitating a state of partial EMT.

**Abstract:**

Epithelial–mesenchymal transition (EMT) and its reversal, mesenchymal–epithelial transition (MET) drive tissue reorganization critical for early development. In carcinomas, processing through EMT, MET, or partial states promotes migration, invasion, dormancy, and metastatic colonization. As a reversible process, EMT is inherently regulated at epigenetic and epigenomic levels. To understand the epigenomic nature of reversible EMT and its partial states, we characterized chromatin accessibility dynamics, transcriptomic output, protein expression, and cellular phenotypes during stepwise reversible EMT. We find that the chromatin insulating protein machinery, including CTCF, is suppressed and re-expressed, coincident with broad alterations in chromatin accessibility, during EMT/MET, and is lower in triple-negative breast cancer cell lines with EMT features. Through an analysis of chromatin accessibility using ATAC-seq, we identify that early phases of EMT are characterized by enrichment for AP-1 family member binding motifs, but also by a diminished enrichment for CTCF binding motifs. Through a loss-of-function analysis, we demonstrate that the suppression of CTCF alters cellular plasticity, strengthening the epithelial phenotype via the upregulation of epithelial markers E-cadherin/CDH1 and downregulation of N-cadherin/CDH2. Conversely, the upregulation of CTCF leads to the upregulation of EMT gene expression and an increase in mesenchymal traits. These findings are indicative of a role of CTCF in regulating epithelial–mesenchymal plasticity and gene expression.

## 1. Introduction

Epithelial–mesenchymal transition (EMT) is a conserved process that alters the differentiation state of a cell in order to drive physiological programs, such as gastrulation and wound healing. During EMT, epithelial cells alter their gene expression and morphology, lose cell–cell contacts, and adopt a mesenchymal-like state [1]. Due to the fact that this process promotes invasion, intravasation, and resistance to anoikis, EMT is implicated in metastatic tumor cell dissemination [2,3,4]. Recent work has contributed to a revised model of metastasis in which the reversal of EMT, mesenchymal–epithelial transition (MET), is necessary for the colonization of cells that arrive at the metastatic site by means of an EMT [2,3,4,5]. Cellular plasticity, which is essential for EMT reversal, is enabled by changes in genes expression and chromatin accessibility.

EMT propels cells through progressive gene expression changes and phenotypic alterations. The hallmark of EMT is the suppression of transcription of genes such as E-cadherin (*CDH1*) and epithelial cell adhesion molecule (*EPCAM*), that can be effected through networks of EMT transcription factor proteins (EMT-TFs) such as SNAIL (*SNAI1*), SLUG (*SNAI2*), ZEB1, TWIST1, SIX1, SOX10, and FOXC2 [6,7,8,9,10]. These transcription regulators are known to act in conjunction with epigenetic regulatory mechanisms, such as the post-translational modification of histone proteins [11,12,13,14] and DNA methylation [15,16]. Further key regulatory mechanisms include alternative splicing, microRNAs, and protein translation [17,18,19,20,21,22,23]. EMT is known to be initiated by microenvironmental signals, such as TGFβ, EGF, hypoxia, and tissue stiffness [24,25,26,27]. Conversely, the loss of such stimuli can trigger MET and the re-establishment of cell–cell contacts, a decrease in migratory traits, and the expression of epithelial-specific transcription factors such as ELF5, GRHL2, and OVOL1/2 [28,29,30].

Diverse partial- or hybrid-EMT states, which co-exhibit epithelial and mesenchymal traits, are reported to be highly plastic, and, when present in tumor cells, efficiently initiate tumor growth, and predict poor patient outcomes [31,32,33,34,35,36,37,38,39,40]. Distinct hybrid EMT states are likely driven by individual EMT-TFs [41,42], which, despite considerable overlap within the EMT-regulatory network, lead to distinct gene expression outcomes [10,43]. Additionally, the propensity of a transcription factor to regulate gene expression can be further modulated by chromatin accessibility at transcription factor binding sites. Local chromatin accessibility, enabled by chromatin remodeling proteins, contort DNA to reveal regulatory sites that can be bound by a suite of transcription factors. These local DNA environments can be further reinforced by the chromatin binding factor CTCF through interactions with cohesin and condensin protein complexes, which jointly create gene expression neighborhoods that can be regulated by similar enhancers, shield regulatory sites, and contribute to broad-scale chromatin organization [44]. CTCF expression, localization, and DNA-binding activity is critical for cellular differentiation [45], yet the role of CTCF in reversible EMT is unclear. Therefore, we conducted phenotypic, transcriptomic, and chromatin-accessibility-focused analyses of cells at progressive stages along the epithelial–mesenchymal plasticity spectrum, as well as a functional assessment of the role of CTCF in epithelial–mesenchymal plasticity.

We characterized the genome-wide dynamics of chromatin accessibility at multiple timepoints during EMT and MET, including the relationship between chromatin accessibility, gene expression, and cell phenotype. By staggering TGFβ exposure and withdrawal, we established a series of stepwise EMT/MET states. Distinct phases of the repression and re-expression of the key epithelial marker E-cadherin were evident in terms of protein localization, protein expression, transcript expression, and locus accessibility. Utilizing the assay for transposase-accessible chromatin with next-generation sequencing (ATAC-seq), we report that EMT imparts a global increase in transposase-accessible chromatin, whereas MET is marked by chromatin compaction. Transcription factor binding site (TFBS) enrichment analysis points to a dynamic engagement of CTCF throughout EMT/MET. Importantly, we find that CTCF knockdown, or a reduction in chromatin-bound CTCF, reinforces epithelial gene expression and phenotypes, and CTCF overexpression stimulates mesenchymal gene expression patterns and increases cell migration. Collectively, our findings indicate that the activation of EMT and MET dramatically reconfigures chromatin accessibility, and that CTCF is a key modulator of epithelial–mesenchymal plasticity by affecting the transcriptomic output of critical EMT genes.

## 2. Materials & Methods

### 2.1. Biological Resources

MCF10A cells were a gift from Dr. Sendurai Mani and were cultured as previously described [46]. HMLE cell lines were also a gift from Dr. Sendurai Mani and were cultured as described previously [43]. Cells were plated at 10,000 cells/cm^2^ and passaged every other day to maintain consistent densities. For TGFβ treatment, media was supplemented with 5 ng/mL recombinant human TGFβ-1 (R&D Systems, Minneapolis, MN, USA; resuspended in 4 mM HCl, 0.1% BSA) and were replenished with every cell passage. MCF7, SKBR3, MDA-MB-231, Hs-578t cell lines (ATCC, Manassas, VA, USA), and HEK-293T cells, which were a gift from Dr. Jason Herschkowitz, were cultured in DMEM media (Corning, Corning, NY, USA) supplemented with 10% FBS (Cytiva, Marlborough, MA, USA), and 1% penicillin/streptomycin (Lonza, Basel, Switzerland). Cells were regularly tested for mycoplasma contamination using PlasmoTest^TM^ Mycoplasma Detection Kit (InvivoGen, San Diego, CA, USA).

### 2.2. Viral Transduction

FUGW-d2GFP-ZEB1 and pHAGE-E-cadherin-RFP plasmids were a gift from Dr. Michael Toneff. The pHAGE-CTCF plasmid was a gift from Gordon Mills & Kenneth Scott (Addgene #116728) and pHAGE-GFP was a gift from Jay Shendure (Addgene #106281). Stable reporter cell lines were generated via viral transfection as described [47]. pTRIPZ-shCTCF (Horizon Discovery, #RHS4696-200764825), pTRIPZ-shCtrl (Horizon Discovery, #RHS4743), pHAGE-GFP, and pHAGE-CTCF were transfected alongside pCMV-Δ8.2 and pCMV-VSVG using FuGENE HD transfection reagent (Promega, Madison, WI, USA) and DMEM. Viral supernatant was clarified using a 0.45 μm filter before addition to HEK-293T cells. FUGW-d2GFP-ZEB1-GFP^+^ were labeled and GFP^+^ pHAGE cells were isolated using FACSMelody^TM^ Cell Sorter (BD Biosciences, Franklin Lakes, NJ, USA). pTRIPZ-expressing cells were selected with 0.5 µg/mL puromycin.

### 2.3. Cell Fractionation

Subcellular protein fractionation was performed on 5 × 10^6^ cells using the Subcellular Protein Fractionation Kit for Cultured Cells (Thermo Fisher Scientific, Waltham, MA, USA) following the manufacturer’s protocol. Ten µg total protein was loaded into each well for Western blotting.

### 2.4. Western Blotting

Cells were harvested and resuspended in RIPA buffer (Alfa Aesar, Haverhill, MA, USA) supplemented with protease and phosphatase inhibitors (Thermo Fisher Scientific, Waltham, MA, USA) and incubated in ice for 60 min. Lysed cells were centrifuged at 15,000 rcf for 20 min at 4 °C and the supernatant was isolated. Protein concentrations were determined using a bicinchoninic acid assay (Thermo Fisher Scientific, Waltham, MA, USA).

Proteins were separated by a 12% SDS/PAGE gel, transferred onto a 0.45 µm PVDF membrane (Thermo Fisher Scientific, Waltham, MA, USA), and probed with the appropriate antibodies. Chemiluminescence signal was obtained using ECL^TM^ Prime (Cytiva, Marlborough, MA, USA), and blot images were captured using the ChemiDoc^TM^ Imaging System (Bio-Rad, Hercules, CA, USA).

### 2.5. Flow Cytometry

At the conclusion of the TGFβ time course, cells were counted and resuspended in 500 µL 1% FBS (Cytiva, Marlborough, MA, USA) in PBS with anti-E-cadherin BB700 (BD Biosciences, Franklin Lakes, NJ, USA; #745965, 1:100), anti-CD44 BV421 (BioLegend, San Diego, CA, USA; #562890, 1:100), and anti-CD24 (BioLegend, San Diego, CA, USA; #311104, 1:100), and incubated on ice for 90 min. Following incubation, cells were pelleted and washed twice with 1% FBS in PBS and subjected to flow cytometry using a FACSMelody^TM^ (BD Biosciences, Franklin Lakes, NJ, USA).

### 2.6. Reagents

The following antibodies (in 5% milk in TBST) were used for Western blotting: anti-E-cadherin (#14472, monoclonal mouse, 1:1000, Cell Signaling Technologies, Danvers, MA, USA), anti-N-cadherin (#BDB610920, monoclonal mouse, 1:1000, BD Biosciences, Franklin Lakes, NJ, USA), anti-Slug (#9585, monoclonal rabbit, 1:500, BD Biosciences, Franklin Lakes, NJ, USA), anti-CTCF (#07-729, polyclonal rabbit, 1:1000, Millipore Sigma, Burlington, MA, USA), anti-BORIS/CTCFL (PA5-97639, polyclonal rabbit, 1:1000, Thermo Fisher Scientific, Waltham, MA, USA), anti-SMC3 (#5696, monoclonal rabbit, 1:1000, Cell Signaling Technologies, Danvers, MA, USA), anti-YY1 (#46395S, monoclonal rabbit, 1:1000, Cell Signaling Technologies, Danvers, MA, USA), anti-ZEB1 (21544-1-AP, polyclonal rabbit, 1:2000, ProteinTech), anti-Vimentin (#103661-AP, 1:2000, ProteinTech), anti-Actin (#612656, monoclonal mouse, 1:2000, BD Biosciences, Franklin Lakes, NJ, USA), anti-H3K27me3 (#9733S monoclonal rabbit, Cell Signaling Technologies, Danvers, MA, USA), anti-H3K4me3 (#9751S, monoclonal rabbit, Cell Signaling Technologies, Danvers, MA, USA), anti-H3 (#4499S, monoclonal rabbit, Cell Signaling Technologies, Danvers, MA, USA), anti-RFP (#MA5-15257, 1:2000, Thermo Fisher Scientific, Waltham, MA, USA), anti-rabbit HRP-linked IgG secondary (#7074S, 1:2000, Cell Signaling Technologies, Danvers, MA, USA), and anti-mouse HRP-linked IgG secondary (#926-80010, 1:2000, Li-Cor, Lincoln, NE, USA).

### 2.7. RNA Extraction and qPCR

RNA was extracted from cell cultures using TriZol Reagent (Thermo Fisher Scientific, Waltham, MA, USA) and isolated following manufacturer guidelines. A total of 500 ng of RNA was used for cDNA synthesis. TaqMan Gene Expression primers (Thermo Fisher Scientific, Waltham, MA, USA) were used for miR-203, miR-200c, and sno-U6. MicroRNA quantitative PCR analyses were performed using TaqMan Gene Expression Master Mix (Applied Biosystems), normalizing to sno-U6. PowerUp SYBR Green Master Mix (Thermo Fisher Scientific, Waltham, MA, USA) and a QuantStudios5 real-time PCR machine (Thermo Fisher Scientific, Waltham, MA, USA) were used for quantitative PCR analyses with four technical replicates per biological replicate, normalizing to *ACTB*. Signal was quantified and normalized using QuantStudios5 software (version 1.5.1) and analyzed using Prism (version 8.4.3, Graphpad, San Diego, CA, USA).

### 2.8. Mammosphere Assay

At the conclusion of the time course, 5000 cells were plated in low-attachment 96-well plates containing 100 µL MEGM media (without BPE) (Lonza, Basel, Switzerland, CC-3150), 20 ng/mL FGF (Sigma Aldrich, St. Louis, MO, USA), 10 ng/mL EGF (Sigma Aldrich, St. Louis, MO, USA), 4 µg/mL heparin (Sigma Aldrich, St. Louis, MO, USA), and 1% methylcellulose. Twenty-five µL fresh mammosphere media were added every third day. Spheres were allowed to form for 14 days.

### 2.9. Wound Healing Assay

Cells were plated to reach 100% confluency at the end of time course. pTRIPZ-shCTCF and pTRIPZ-shCtrl cells were pretreated with 3 µg/mL doxycycline two days before wounding with a p200 tip. Measurements were made using Nikon (Minato City, Tokyo, Japan) NIS Elements Imaging Software (version 4.5) and analyzed using Prism (version 8.4.3, Graphpad, San Diego, CA, USA).

### 2.10. RNA-Seq Library Preparation and Sequencing

RNA was extracted from cell cultures using TriZol Reagent (Thermo Fisher Scientific, Waltham, MA, USA) and isolated following manufacturer guidelines. Libraries were prepared using TruSeq Stranded mRNA Library Prep Kit (Illumina, San Diego, CA, USA). Sequencing was performed by Macrogen (Seoul, South Korea) and Novogene (Sacramento, CA, USA).

### 2.11. Identification of Differentially Expressed Genes

We used Salmon [48] to quantify the expression of transcripts from FASTQ files for both the CTCF overexpression and knockdown experiments. We indexed the reads using the GRCh38 human genome with the following options: —threads 8—gcBias—validateMappings. Quantifications were imported to DESeq2 (v1.32.0) using the tximeta (v1.10.0) package in R (v4.1.1) [49,50]. DESeq2 was used to identify differentially expressed genes in each of the experimental conditions. We considered two comparisons: differential expression of pHAGE-CTCF compared to pHAGE-GFP, and shCTCF compared to shCtrl with doxycycline only.

### 2.12. EMT Score Calculation

The EMT scores were calculated utilizing the 76-gene expression signature reported [51] and the metric mentioned based on that gene signature [52]. For each sample, the score was calculated as a weighted sum of 76 gene expression levels, and weights were measured based on the correlation of a particular gene with *CDH1* expression. The scores were standardized for all of the samples in the dataset by subtracting the mean across samples so that the global mean of the score was zero. Negative scores calculated using this method can be interpreted as mesenchymal phenotype and the positive scores as epithelial.

### 2.13. ATAC-Seq Library Preparation and Sequencing

ATAC-seq libraries were generated as described [53]. Briefly stated, 50,000 cells were centrifuged, resuspended in 50 µL lysis buffer (10 mM Tris, 10 mM NaCl, 3 mM MgCl_2_, and 0.1% IGEPAL CA-630), and centrifuged at 500× *g* for 10 min at 4 °C. The pellet was resuspended in transposase reaction mix (25 µL 2× TD buffer, 2.5 µL transposase (Nextera DNA sample preparation kit, Illumina), and 22.5 µL water, and incubated at 37 °C for 30 min. Tagmented DNA was purified using MinElute PCR Purification Kit (Qiagen, Hilden, Germany) per manufacturer’s instructions. DNA libraries were PCR-amplified using Nextera DNA Sample Preparation Kit (Illumina, San Diego, CA, USA) using the following PCR conditions: 98 °C for 30 s, then thermocycling for 98 °C for 10 s, 63 °C for 30 s, and 72 °C for 1 min for 12 cycles, followed by 72 °C for 5 min. PCR products were size-selected for 200 to 800 base pair fragments using SPRI-Select Beads (Beckmann-Coulter, Brea, CA, USA). ATAC-seq reads were paired-end sequenced using an Illumina NextSeq500 (BSWRI Core Facility, Dallas, TX, USA).

### 2.14. Computational Resources: ATAC Peak Abundance and Motif Analysis

Due to the similarities of both ends, one paired-end read was used for analysis. Adapter sequences were removed using cutadapt (version 1.16.6) and reads were cropped to 30 bp with Trimmomatic (version 0.36.6). ATAC-seq reads were aligned to *hg19* using Bowtie2 (version 2.3.4.2). Mitochondrial, unmapped and random contigs, and ChrY reads were excluded using samtools (version 1.9) [54] to generate filtered bam files for downstream analysis.

Tag directories were generated in HOMER from filtered bam files [55]. Peaks from each replicate were called separately using HOMER ‘findPeaks -style dnase -o auto’. Then, replicate peaks were merged by calling ‘mergePeaks -d 300’ to determine common peaks. Common peaks were used for downstream analyses.

For Pearson correlation analyses, combined tag directories containing both replicates were used to quantify average peak score. Common merged peaks from each replicate were merged with other time course conditions by calling ‘mergedPeak -d 300’. Time course peak files were annotated to *hg19* by calling “annotatePeaks.pl hg19 -size 300 -log-d”. Peak scores for each condition were quantile-normalized by the preprocessCore package (version 1.44.0) in R (version 3.5.1). Quantile-normalized Pearson correlation scores were generated by R Base and visualized by the pheatmap R package (version 1.0.12).

Known motif searches were performed using HOMER [55] for 50 base pair regions, excluding masked genomic regions by calling “findMotifsGenome.pl hg19 -size 50 -mask”. Motifs with *p*-values > 10^−12^ were discarded. Plots representing the number of significant motifs (*p*-value ≤ 10^−12^) and their enrichment were generated using the ggplot2 R package (version 3.2.0).

To identify the differential peaks, the HOMER function getDifferentialPeaks was used. This function was used to quickly identify which peaks contain significantly more tags in the target experiment relative to the background experiment. Here, in each pairwise comparison, vehicle sample was considered as the background sample. The differential peaks were identified based on default parameters; i.e., peaks that have 4-fold more tags (sequencing-depth independent) and a cumulative Poisson *p*-value less than 0.0001 (sequencing-depth dependent). Next, we annotated these differential peaks to genes and obtained the distance of these peaks from the gene transcription start site (TSS). After obtaining the annotated peaks, we looked at the peak distance from TSS together with the fold change in the expression of respective genes. The fold change for a given gene was calculated by simply taking the ratio of expression values in the two samples.

For the GIGGLE analysis, ATAC-seq BED files were normalized using BEDTools, and peaks were called using MACS2. Input peak files were queried by GIGGLE [56] against ~47,000 CistromeDB compiled genome interval files, and chromatin accessibility regulators with significant feature overlap to queried files, as quantified by similarity scores, were identified [57,58].

### 2.15. GSEA and Enrichment Analysis

Peak similarities were identified using mergePeaks in HOMER and annotated to *hg19* to produce the Entrez IDs for the gene promoters nearest to peaks. GSEA analysis for Molecular Signatures Database (MSigDB) hits was performed for specific peak groups, and for sets of differentially expressed genes using WebGestalt [59]. We controlled for FDR and considered significance at an FDR < 0.05. As a result of the abundance of MSigDB hits from ATAC-seq data, our analysis was limited to either unbiased hallmarks-only gene sets [60] or breast and mammary-specific gene sets (containing keywords “breast” or “mammary”).

We quantified overlap between genes near CTCF motifs and differentially expressed genes using the presence of the same ID in each gene set. The significance of overlap was calculated using the hypergeometric test.

### 2.16. ChIP-Seq Data Pre-Processing

ChIP-seq bed data files from Fritz et al. [61] were downloaded using SRA Toolkit (version 2.10.5). Before permutation analysis, Bedtools was used to filter out any regions overlapping a list of previously described ENCODE blacklisted and assembly gap regions for ChIP-seq [62].

### 2.17. Peak Enrichment Analysis

For the provided CTCF ChIP-seq file, we calculated base pair overlap between the mark and the ATAC peaks. We used a permutation-based technique to determine whether the observed amount of base pair overlap was more than expected by chance. We calculated an empirical *p* value for the observed amount of overlap by comparing to a null model obtained by randomly shuffling length-matched regions throughout the genome and calculating the amount of base pair overlap in each permutation. Where relevant, the *p*-values were adjusted for multiple testing using the Bonferroni correction.

### 2.18. Oligos

The primers used for PCR amplification in this study can be found in Supporting Materials.

### 2.19. Data Availability

The ATAC and RNA sequencing data generated in this work have been deposited in NCBI’s Gene Expression Omnibus and are accessible though GEO series accession number GSE145851. ATAC-seq files are accessible through the UCSC genome browser [https://genome.ucsc.edu/s/kelsey_johnson1/Reversible%20EMT%20ATAC%2Dseq%20peaks].

## 3. Results

### 3.1. TGFβ Induces EMT Phenotypes

**Figure 1:**
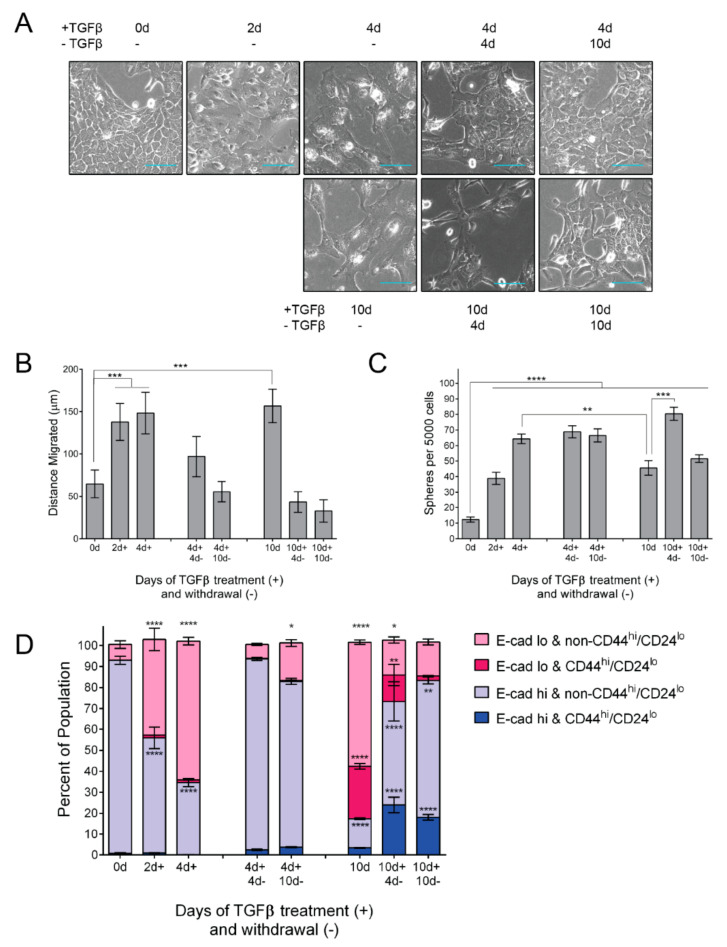
TGFβ treatment and withdrawal elicits phased cell biological changes indicative of EMT-MET. (**A**) Brightfield photomicrographs of MCF10A cells at indicated durations; TGFβ used at 5 ng/mL; scale bars = 100 µm. (**B**) Migration capacity was determined using a scratch-wound healing assay. MCF10A cells were treated as indicated prior to re-plating, at confluency, in media lacking TGFβ. The change in average gap length after 10 hours is reported. Error bars indicate s.e.m. (*n* = 6). Statistical significance was tested using a one-way ANOVA followed by comparison of each mean to untreated cells using a Dunnet correction for multiple hypothesis testing. (**C**) Mammosphere formation capacity of cells treated as indicated. Following the conclusion of the indicated treatments, TGFβ-treated and -withdrawn cells were subjected to mammosphere-promoting conditions for 10 days, with no exogenous TGFβ, and mammospheres ≥ 50 µm were counted. Error bars indicate s.d. (*n* = 6). Statistical significance was tested using a one-way ANOVA followed by comparison of each mean to untreated cells using a Dunnet correction for multiple hypothesis testing. (**D**) FACS profiling of CD44, CD24, and surface E-cadherin in MCF10A cells treated as indicated. Cells were categorized as either E-cadherin-high (blue) or -low (pink) and either CD44^hi^/CD24^lo^ (lighter shading) or non-CD44^hi^/CD24^lo^ (darker shading). Error bars indicate s.d. (*n* = 3). Statistical significance was tested using a one-way ANOVA followed by comparison of each mean to untreated cells using a Dunnet correction for multiple hypothesis testing. * *p* ≤ 0.05, ** *p* ≤ 0.01, *** *p* ≤ 0.001, **** *p* ≤ 0.0001.

MCF10A human epithelial cells, derived from spontaneously immortalized fibrocystic mammary tissue [63], are widely utilized as model for epithelial–mesenchymal plasticity [23,64,65,66]. To characterize progressive epithelial–mesenchymal transition (EMT), we subjected MCF10A cells to TGFβ treatment and withdrawal for varying durations [67,68]. The cells’ characteristic epithelial morphology was lost after 2 days of TGFβ treatment, while a mesenchymal spindle-like morphology emerged at 4 days of TGFβ treatment (short-term TGFβ) (Figure 1A). The continued treatment of TGFβ for up to 10 days resulted in an elongated morphology. Ten days of withdrawal was necessary for cells subjected to long-term TGFβ (10 days of TGFβ) to resolve back to an epithelial morphology (Figure 1A).

We next assessed phenotypic alterations associated with EMT. As expected, cells treated with TGFβ demonstrated a greater migratory capacity than control cells (Figure 1B). This effect is reversible, as TGFβ withdrawal suppressed the migratory capacity (Figure 1B). Likewise, the mammosphere formation capacity, an indicator of stem-like properties imparted through EMT [69], is elevated in TGFβ-treated cells (Figure 1C). These results demonstrate an enrichment in stem-like properties in partial-EMT states.

To further understand the progressive changes in the stem-like phenotype, we next assayed for the cell surface expression of E-cadherin, an indicator of the epithelial phenotype, as well as CD44 and CD24, for which the combination of CD44^hi^/CD24^lo^ is indicative of a stem cell-rich subpopulation [70]. As expected, TGFβ treatment decreases the proportion of surface E-cadherin-positive cells (shown in blue) from 93.0% to 34.6% in short-term (4 days TGFβ) treated cells and to 17.5% in long-term (10 days TGFβ) treated cells (Figure 1D). We were curious about the durability of these observations, so we withdrew the short-term TGFβ treated cells from TGFβ and assessed surface E-cadherin. The withdrawal from short-term TGFβ treatment elicited a rapid recovery of surface E-cadherin (93.7% after 4 days withdrawal), whereas the long-term TGFβ treatment resulted in a slower recovery of surface E-cadherin expression (83.2% after 4 days withdrawal) (Figure 1D). Interestingly, long-term TGFβ induces a CD44^hi^/CD24^lo^ population within the surface E-cadherin^hi^ population, which comprises 3.3% of the total population, compared to 0.4% of untreated cells, (Figure 1D, dark blue). TGFβ withdrawal facilitates an expansion of this population, which increases to 24.0%, and then decreases to 18.1%. The persistence of this population indicates a potential source of stemness in cells undergoing MET. Overall, our time course reveals the asymmetrical acquisition and resolution of EMT phenotypes resulting in distinctions in morphology, E-cadherin localization, and migratory capacity.

### 3.2. TGFβ Induces Gene Expression Dynamics

**Figure 2:**
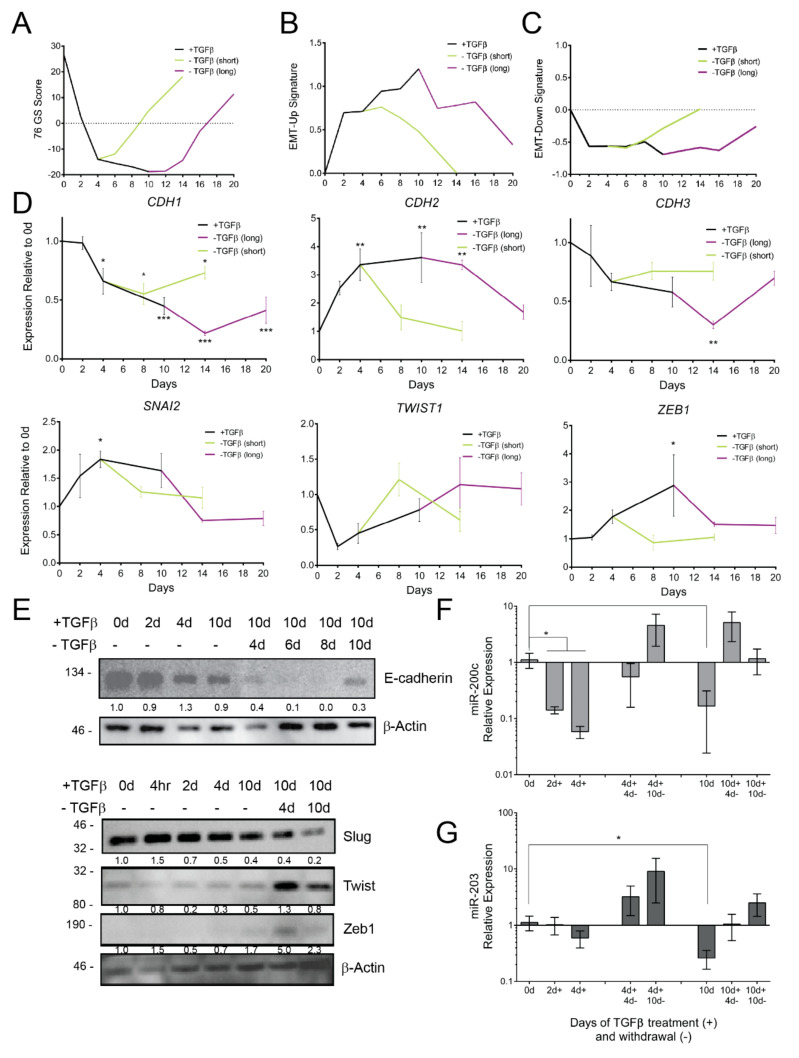
Gene expression dynamics during TGFβ treatment and withdrawal. RNA-seq data were analyzed with regard to expression of a (**A**) 76-gene epithelial metric [51], (**B**) core EMT-Up and (**C**) core EMT-Down gene signatures [43]. (**D**) mRNA expression of select epithelial and mesenchymal genes was determined using qPCR, normalized to untreated cells and *ACTB*, and is represented as mean and s.d. (*n* = 3). Statistical significance was tested using a one-way ANOVA followed by comparison of each mean to untreated cells using a Dunnet correction for multiple hypothesis testing. (**E**) Western blot for epithelial (top) and EMT-TFs (bottom) in MCF10A cells treated with TGFβ for the indicated durations. Expression of (**F**) miR-200c and (**G**) miR-203 was normalized to sno-U6 and is represented as mean and s.d. Statistical significance was tested using a one-way ANOVA followed by comparison of each mean to untreated cells using a Dunnet correction for multiple hypothesis testing (*n* = 4). * *p* ≤ 0.05, ** *p* ≤ 0.01, *** *p* ≤ 0.001.

We next evaluated changes in EMT-associated gene expression by RNA-seq, qPCR, and western blotting. To measure broad changes in gene expression, we performed RNA-seq and scored the gene expression based on the epithelia-specific 76-gene score (76GS) [71]. Positive 76GS scores correspond to an epithelial gene expression pattern. TGFβ treatment progressively suppresses the 76GS score until TGFβ withdrawal, whereupon the score progressively recovers (Figure 2A), irrespective of the initial duration of TGFβ treatment. Additionally, we assessed the progression of EMT-related gene expression using a mammary-cell-specific EMT signature [43]. Gene expression data are plotted for 50 genes that were previously shown to be commonly upregulated (EMT-Up) or down-regulated (EMT-Down) by multiple EMT-TFs in HMLE mammary cells [43]. In our model, EMT-Up genes increase their expression continuously throughout the time course (Figure 2B), whereas EMT-Down gene expression reaches a minimum level after 2 days of TGFβ treatment (Figure 2C). We further analyzed the mRNA expression of specific genes through qPCR. *CDH1* (E-cadherin) and *CDH3* (P-cadherin), a proposed partial-EMT marker [72], show progressive downregulation, which is durable through TGFβ withdrawal (Figure 2D). *CDH2* (N-cadherin) and EMT-TF *SNAI2* gene expression show an immediate responsiveness to TGFβ treatment and withdrawal (Figure 2D). On the other hand, EMT-TF *ZEB1* gene expression shows a delayed upregulation, whereas *TWIST1* shows no significant change (Figure 2D).

Many EMT-related genes are regulated by translation and are efficacious as proteins [23,73]; therefore, we assessed changes in protein expression for EMT markers and EMT-TFs. We observe that TGFβ suppresses E-cadherin and increases the expression of EMT-TFs Slug, Twist, and ZEB1 (Figure 2E)**.** The expression of the EMT-TF Slug mRNA and protein expression peaked early in TGFβ treatment, whereas the expression of Twist and ZEB1 did not reach its highest until later in the long-term TGFβ treatment model (Figure 2E). Indeed, the highest expression of Twist and ZEB1 was not observed until further into EMT induction, just after TGFβ withdrawal had begun, which is consistent with recently published data by Deshmukh et al. [68]. This successive enrichment of EMT-TFs may be indicative of a progressive activation of EMT-TFs wherein partial EMT is likely driven by early EMT-TFs, such as Slug, as had been predicted through a modeling approach [41].

We next examined additional facets of *CDH1* promoter and *ZEB1* 3′UTR regulatory dynamics in our model. MCF10A cells were transduced with the *CDH1* promoter linked to an RFP-encoding gene, facilitating promoter activity tracking at the single cell level [47]. Commensurate with gene expression data, the *CDH1* promoter reporter remained active throughout the short-term TGFβ treatment. However, upon extended treatment, reporter-negative cells outnumbered reporter-positive cells, indicating promoter-induced repression (Supplementary Figure S1). Notably, the repression of the *CDH1* promoter was durable despite ten days of withdrawal. In order to measure the post-transcriptional regulation of *ZEB1*, we used a 3′UTR activity assay [47]. MCF10A cells were transduced with a GFP-linked *ZEB1* 3′UTR reporter (GFP-Z1) and were subjected to TGFβ treatment. GFP-Z1 expression within GFP-positive cells became elevated at two days of TGFβ treatment (Supplementary Figure S1), agreeing with the detectable ZEB1 protein and increase in *ZEB1* mRNA. Unlike *CDH1*, the *ZEB1* 3′UTR reporter readout returned to baseline upon TGFβ withdrawal (Supplementary Figure S1). Epithelial-specific microRNAs-200c (miR-200c) and -203 (miR-203) are repressed during EMT and have been reported to target *ZEB1* and *SNAI2* [74,75]. As expected, miR-200c showed a significant decrease in expression following 2 days of TGFβ treatment, whereas the suppression of miR-203 did not reach significance until completions of long-term TGFβ treatment (Figure 2F,G). TGFβ withdrawal elicited the re-expression of both microRNAs (Figure 2F,G). Altogether, these data are consistent with an early (2 days of TGFβ) suppression of miR-200c, which may alleviate the repression of *ZEB1* mRNA and upregulate the ZEB1 protein.

### 3.3. EMT and MET-Induced Changes in Accessible Chromatin Regions

EMT is accompanied by reversible changes in the epigenome [46]. Chromatin accessibility orchestrates dynamic gene expression by exposing or hiding genomic regulatory elements. The systematic coordination of genomic elements produces distinct epigenomic states [76]. To uncover the epigenomic basis of TGFβ-driven reversible EMT, we performed the assay for transposase-accessible chromatin with next-generation sequencing (ATAC-seq) [53].

**Figure 3:**
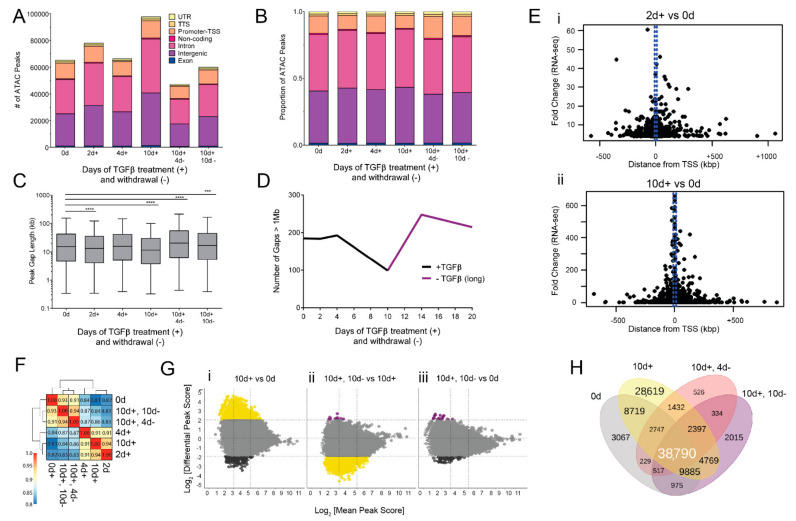
Dynamics of EMT- and MET-associated chromatin accessibility. (**A**) Number and (**B**) proportion of ATAC-seq peaks according to function of the genomic region and duration of TGFβ treatment and withdrawal. (**C**) Gap length distribution between ATAC peaks. Center line represents mean, box edges represent 25th and 75th percentiles, and whisker ends represent 5th and 95th percentiles. Data were analyzed using two-way ANOVA using Tukey’s multiple comparison test vs. 0 d. *** *p* ≤ 0.001, **** *p* ≤ 0.0001. (**D**) Number of gaps between peaks that exceed 1 Mb, excluding centrosome sequence. (**E**) Scatter plot showing fold-change in gene expression (RNA-seq) vs. distance from TSS for specific genes showing differential peak intensity for (i) 2 d + TGFβ vs. 0 d and (ii) 10 d + TGFβ vs. 0 d timepoints. Each dot represents a peak with at least four-fold more accessibility. (**F**) Pearson’s correlation analysis on quantile-normalized log-transformed ATAC peaks. (**G**) Differential accessibility (log_2_fold change in reads per 300-bp region) among indicated conditions. Colored dots represent peaks with at least four-fold more accessibility at the indicated conditions. Black = 0 d, yellow = 10 d + TGFβ, and purple = 10 d + TGFβ, 10 d-TGFβ. (**H**) Venn diagram representing the overlap between ATAC-seq peaks common among untreated and TGFβ-treated conditions.

The extent of chromatin accessibility is highly dynamic across EMT and MET. Long-term TGFβ treatment was associated with additional ATAC-seq peaks, whereas withdrawal from TGFβ treatment was associated with a diminished number of ATAC-seq peaks (Figure 3A). Dramatically, long-term treatment led to 50% more peaks than untreated cells (175,103 vs. 113,680). This was reversed upon 4 days TGFβ withdrawal, as the number of peaks dramatically decreased to 47,174. Though the number of called peaks fluctuate, the proportion of peaks annotated to untranslated regions (UTR), transcriptional termination sites (TTS), and exons remains stable throughout TGFβ treatment (Figure 3B). However, a greater variation was evident in the proportion of intergenic and promoter-associated peaks that increase or decrease, respectively, in response to TGFβ (Figure 3B). These results suggest a broad effect of TGFβ treatment on chromatin structure.

In order to further determine the genomic distribution of newly accessible chromatin regions, we interrogated the distance, in base pairs, between annotated ATAC peaks. Generally, TGFβ treatment decreases the gap size between peaks (Figure 3C). Long-term TGFβ-treated cells had significantly shorter distances between ATAC-seq peaks (median 11,564 bp) compared to the control (median 15,348 bp). Due to the fact that many of the peaks unique to long-term TGFβ annotate to intergenic and intronic regions, we hypothesized that TGFβ treatment decreased the number of 1 megabase (Mb) regions without a peak—reducing the number of so-called “peak deserts”. We enumerated the number of ATAC-seq peak deserts and found that long-term TGFβ treatment decreases the number of peak deserts from 185 to 99 (Figure 3D). To understand how changes in chromatin accessibility associate with changes in transcription, we plotted the fold change in gene expression against the distance from TSS for peaks with a significantly differential peak intensity between either 2 days TGFβ (Figure 3Ei) or 10 days TGFβ (Figure 3Eii) and the control. As expected, the strongest gene expression fold change values are associated with ATAC-seq peaks nearest to TSSs. Nevertheless, genes with significant changes in chromatin accessibility of up to 500 kb on either side of the TSS also exhibit altered expression (Figure 3E). Overall, these data suggest that EMT increases chromatin accessibility across the genome, with TSS distal and proximal changes affecting gene expression.

Given the diverse transcriptional dynamics observed in EMT-related genes, we next examined chromatin accessibility at select genes. We first compared the peaks within epithelial-specific genes *CDH1* (E-cadherin), *CDH3* (P-cadherin), and *EPCAM* (epithelial cell adhesion molecule). Accessibility at *CDH1* and *CDH3* remained high throughout short-term TGFβ treatment, as indicated by minor changes in peak profiles. These results are consistent with *CDH1* relative expression and total E-cadherin protein expression, suggesting that the lack of chromatin perturbations enabled the maintenance of *CDH1* transcription and E-cadherin expression. However, some *CDH1* and *CDH3* peaks began to diminish following long-term TGFβ, and continued to do so despite TGFβ withdrawal, highlighting a delayed and possibly stable chromatin alteration following EMT (Supplementary Figure S2A). These results contrast with chromatin accessibility at the *EPCAM* promoter, where peaks scores are more tightly associated with initial exposure to TGFβ but fail to return to control levels even after 10 days of withdrawal (Supplementary Figure S2A). This difference in the recovery of *EPCAM* accessibility between short and long-term TGFβ exposure is in concordance with findings from the Scheel lab [77] that show high EPCAM linked with the ability to enter a hybrid EMT state, but low EPCAM linked with an irreversible mesenchymal state.

Accessibility patterns at mesenchymal genes *CDH2*, *VIM*, *FN1*, *ZEB1*, *SNAI1*, and *SNAI2* (Supplementary Figure S2B,C) are more responsive to TGFβ than epithelial genes—increasing in accessibility and peak intensity following TGFβ treatment and resolving to untreated conditions in withdrawal. Whereas the peak density at the promoter proximal cluster remains elevated throughout TGFβ treatment and withdrawal, the peak density at the promoter distal cluster of *FN1* (gray box, Supplementary Figure S2B) corresponds closely to TGFβ exposure. Similarly, *SNAI1* and *SNAI2* both show greater changes in peak height at regions distal to the coding sequence or promoter regions (Supplementary Figure S2C).

### 3.4. Chromatin Changes Occur Early in EMT and Are Mostly Reversible

Next, in order to determine the relationships between timepoints in terms of chromatin accessibility patterns, we calculated a correlation coefficient for each pair of samples. As a result of the differences in peak distribution and score among TGFβ-treated and withdrawn conditions, peaks were merged into 300 bp bins, and peak scores were quantile-normalized prior to correlation with other time points. Expectedly, untreated and TGFβ withdrawn conditions share similarities (R^2^ = 0.93) (Figure 3F). The greatest distinction in chromatin accessibility patterns was between untreated and long-term TGFβ treatment (R^2^ = 0.81) and untreated and 2 days TGFβ (R^2^ = 0.82). The lack of similarity in chromatin peaks between untreated and 2 days of TGFβ suggests that chromatin reprogramming occurs very early in EMT induction, preceding many transcriptomic changes. Interestingly, the strong correlation of chromatin accessibility patterns between 2 and 10 days TGFβ (R^2^ = 0.94) (Figure 3F) suggests that early chromatin accessibility alterations are sustained during EMT induction.

We next asked whether major changes in chromatin accessibility occur at regions with low, moderate, or high accessibility. A mean peak score (MPS) was generated for pairs of samples by averaging the peak scores for those two samples. An MPS below 3 was considered “low accessibility”, whereas an MPS above 5 was considered “high accessibility”. We next determined the differential peak score (DPS) for each pair of samples to quantify their change in accessibility. We observed that major changes in accessibility (defined as a DPS above 2 or below −2) occur primarily at low and moderately accessible regions (Figure 3G) but can occur at highly accessible peaks. A major increase in the accessibility at strong peaks is evident when comparing the long-term TGFβ treatment to untreated cells (Figure 3Gi, yellow). This is followed by a major decrease in the accessibility at strong peaks when comparing long-term TGFβ treatment to cells that have undergone a full TGFβ withdrawal (Figure 3Gii, yellow). These differential peak scores are no longer evident when comparing the control to fully withdrawn cells (Figure 3Giii). These data indicate that long-term TGFβ treatment confers a strong increase in accessibility at highly accessible regions, which are likely to directly regulate gene expression, but also at low accessibility regions, which may be more indicative of transcriptional noise.

To better understand the phased implementation of EMT-associated chromatin accessibility, we analyzed common ATAC-seq peaks among TGFβ treatment schemes. As expected, many chromatin accessibility regions in untreated cells are retained throughout the time course (*n* = 38,790) (Figure 3H). However, TGFβ treatment also led to ATAC-seq peaks that either faded after TGFβ withdrawal (*n* = 28,619) or were retained throughout TGFβ withdrawal (*n* = 2397) (Figure 3H).

To derive the functional implications of these chromatin accessibility regions, we performed gene set enrichment analysis (GSEA) for the annotated genes closest to differential ATAC-seq peaks [77]. Among the unique long-term TGFβ peaks, the Molecular Signatures Database (MSigDB) revealed an enrichment of gene sets involved in hallmark EMT (*p* = 5.5 × 10^−11^), TNFα signaling (*p* = 7.0 × 10^−13^), hypoxia (*p* = 2.75 × 10^−6^), and TGFβ signaling (*p* = 0.00017). This enrichment diminishes following TGFβ withdrawal. We were also interested in the overall function of the genes near persistent EMT peaks and MSigDB hits revealed the enrichment of genes involved in EMT (*p* = 1.2 × 10^−6^), apical junction assembly (*p* = 0.0010), and mammary stemness genes (*p* = 1.8 × 10^−10^) (Supplementary Figure S3).

### 3.5. Dynamic Transcription Factor Engagement during EMT and MET

**Figure 4:**
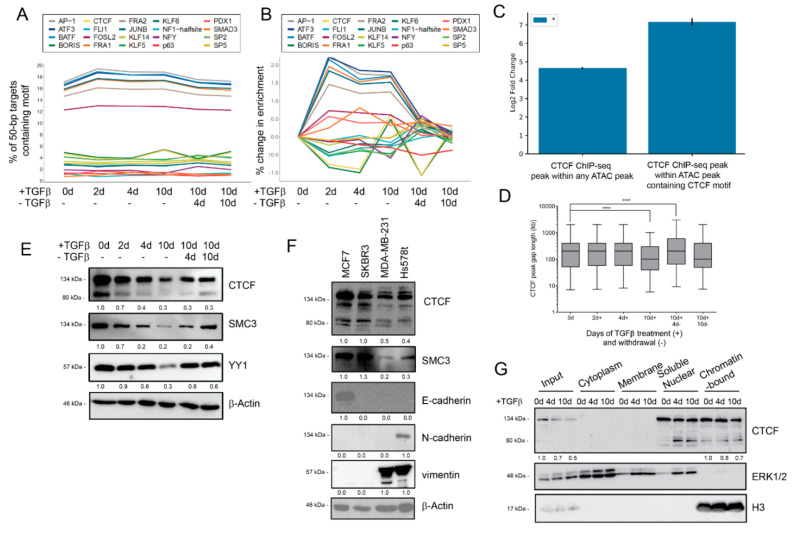
EMT-driven TFBS accessibility and CTCF expression dynamics. (**A**) Motif enrichment by percentage of 50-bp targets containing motif of the top 20 differentially enriched motifs. (**B**) Motif enrichment percentage change, compared to 0 d, of top 20 differentially enriched motifs. (**C**) The overlap between ATAC-seq peaks (in total, or with CTCF motifs) and CTCF ChIP-seq peaks from Fritz et al. [61] was compared to a random distribution model. The observed amount of base pair overlap between the all ATAC-seq peaks and ChIP-seq peaks is 2,758,444 bp, whereas the expected amount of overlap based on our null distribution is 110,000.50 bp (s.d. = 4894.19 bp). For the ATAC peaks with a CTCF binding motif, the enrichment is greater (f.c. = 143, *p* = 0.001). We observe 1,728,041 bp, but expect 12,083.21 bp with a randomly distributed set of genomic regions (s.d. = 1607.12 bp) One thousand permutations were used to generate a *p*-value, which was corrected using Bonferroni method. * *p* ≤ 0.05. (**D**) Gap length distribution (in bp) between ATAC peaks containing CTCF motifs (left). Center line represents mean, box edges represent 25th and 75th percentiles, and whisker ends represent 5th and 95th percentiles. Data were analyzed using two-way ANOVA using Tukey’s multiple comparison test, statistical comparisons shown in table to the right of graph, **** *p* ≤ 0.0001. (**E**) Western blot for CTCF binding partners SMC3 and YY1 during long-term TGFβ treatment and withdrawal model. (**F**) Western blot for CTCF and BORIS protein expression in indicated breast cancer cell lines. (**G**) Western blot for CTCF expression in specific subcellular fractions in MCF10A cells treated with 4 or 10 days TGFβ in comparison to untreated control. H3 used as reference for quantification.

We next tested the hypothesis that TGFβ modulates the enrichment of transcription factor binding sites (TFBS) at accessible regions to enable partial or full EMT. Under the assumption that TF binding can protect a motif from being cleaved by Tn5 transposase, the motif enrichment analysis of ATAC sequences can predict active transcription factors binding at specific EMT/MET timepoints. Using HOMER motif discovery analysis, we limited our analysis to global TFBS, so we segmented the peaks to narrow 50 bp regions and identified the top 20 differentially enriched TFBSs in comparison to untreated conditions [55]. TGFβ increases the enrichment of AP-1 (17.1% in untreated, 18.9% in short-term, and 18.8% in long-term) and SMAD3 (0.72% in untreated, 1.53% in short-term, and 1.04% in long-term) binding motifs and their enrichment resolves to baseline levels during TGFβ withdrawal (Figure 4A). To further confirm the level of SMAD protein binding activity in our time course, we measured the extent of similarity between the pattern of ATAC-seq peaks and curated patterns of genomic occupancy established for DNA-interacting factors using GIGGLE analysis [56]. Limiting our comparisons to genome interval files derived from mammary or breast cancer cells, we observed that similarity scores for SMAD2/3 occupancy patterns diminished over the time course of treatment (Supplementary Figure S4A). Indeed, the SMAD2/3 occupancy patterns yielded the highest similarity scores for 2 days and 4 days of TGFβ treatment, but not for 10 days of TGFβ (Supplementary Figure S4A). Patterns associated with the dimeric AP-1 transcription factor family (FOS, JUN) also yielded high similarity scores for 2 days of TGFβ (Supplementary Figure S4B–E), confirming the HOMER analysis (Figure 4A).

Because of the large range in TFBS enrichment amongst motifs, we also calculated the motif percentage difference from untreated cells (Figure 4B). While the enrichment of most motifs increased following TGFβ treatment, we observed that CTCF (also known as CCCTC binding factor) and BORIS (also known as CTCFL) motif enrichment was diminished during the EMT/MET time course, with the notable exception of long-term TGFβ cells. Indeed, at 2 and 4 days of TGFβ treatment, CTCF and BORIS motif enrichment exhibited the strongest decline for any TFBS motif. TGFβ withdrawal restores these motifs to near-untreated levels. Interestingly, the continued treatment of TGFβ leads to an enrichment of CTCF and BORIS binding motifs. This contrast between short- and long-term TGFβ suggests additional chromatin re-organization between partial and near-complete EMT states may be reinforced by long-range chromatin organization proteins, such as CTCF and/or BORIS.

CTCF and BORIS bind to similar cytosine-rich DNA binding motifs and exhibit some overlapping regulatory functions in relation to establishing topologically associated chromatin domains, regulating genetic imprinting, and modulating gene expression neighborhoods [78,79]. Given the unusual motif enrichment pattern, we next confirmed whether ATAC peaks were considered to be enriched for the CTCF/BORIS motif overlap with validated CTCF binding sites based on available ChIP-seq data in MCF10A cells by Fritz et al., 2017 [61]. We observed a 143-fold enrichment for validated CTCF binding sites in our peaks with the CTCF motif (*p* = 0.001), compared to just a 25-fold enrichment for validated CTCF binding sites in the set of all ATAC peaks (Figure 4C). These data support the notion that the peaks with a predicted CTCF binding site correspond to *bona fide* CTCF-bound sites. The average distance between ATAC peaks with CTCF binding motifs remained stable during the initial phases of TGFβ treatment but dropped significantly at long-term TGFβ treatment, indicating that newly formed ATAC peaks with CTCF binding motifs are not clustering near existing peaks. Upon TGFβ withdrawal, the average distance between ATAC peaks with CTCF binding motifs stabilized back to untreated levels (Figure 4D). As with the increase in general chromatin accessibility (Figure 3C), the change in the distance between accessible regions containing CTCF motifs was consistent with the novel engagement of CTCF bindings sites across the genome, rather than in clusters. Overall, these data reveal that the ATAC peaks containing CTCF motifs align to validated CTCF binding sites and suggest that different CTCF/BORIS motifs may be engaged at various stages of reversible EMT.

Given the loss of CTCF binding sites from accessible chromatin regions during short- but not long-term TGFβ treatment, we determined how CTCF and/or BORIS protein expression was altered over a time course of EMT/MET. Overall, TGFβ treatment suppresses the expression of CTCF protein, whereas TGFβ withdrawal restores expression (Figure 4E). Remarkably, the expression of known CTCF binding partners SMC3 (a member of the cohesin complex) [80] and YY1 (a DNA binding protein that forms enhancer-associated complexes with CTCF) [81] is suppressed during TGFβ treatment in MCF10A cells (Figure 4E), concurrent with CTCF. BORIS, however, exhibited a less dynamic expression pattern (Supplementary Figure S5A). We validated these findings in TGFβ-treated HMLE mammary epithelial cells (Supplementary Figure S5B) and additionally found that mesenchymal breast cancer cell lines MDA-MB-231 and Hs578t cells have a lower CTCF protein expression than epithelial breast cancer cell lines MCF7 and SKBR3 (Figure 4F). Given the dynamic change in CTCF, but not BORIS, we decided to probe the putative involvement of CTCF in reversible EMT and mesenchymal phenotypes.

Considering the TGFβ-driven loss of CTCF motif enrichment in ATAC-seq peaks, we next determined if TGFβ-driven CTCF protein loss affected the nuclear and chromatin localization of CTCF. We isolated specific subcellular fractions following TGFβ treatment and compared the enrichment of CTCF in specific fractions. Diminished CTCF total protein was most evident in the input fraction but was also observed in soluble nuclear and chromatin-bound fractions for TGFβ-treated MCF10A cells (Figure 4G) and in EMT-positive breast cancer cell lines (Supplementary Figure S5B). Although CTCF protein expression is repressed in nuclear fractions, we observe that the CTCF protein is stably expressed with chromatin fractions in long-term TGFβ conditions, which may explain the increase in the CTCF binding motif enrichment in ATAC-seq peaks at that timepoint (Figure 4B). Surprisingly, TGFβ treatment increases the proportion of the smaller ~80 kDa CTCF isoform, CTCF-s, in nuclear fractions (Figure 4G). CTCF-s is known to antagonize the binding of full-length CTCF, thus altering the organization of chromatin looping [80]. This smaller isoform is also evident in breast cancer cell lines and in HMLE cells exposed to TGFβ (Supplementary Figure S5B). These data indicate that the expression, localization, and motif exposure of CTCF and other insulator-associated proteins are altered depending on the EMT/MET state.

We next determined if there were broad functional patterns for genes adjacent to accessible CTCF motifs. Comparing long-term TGFβ treatment and withdrawal, most genes adjacent to CTCF motifs were shared amongst the three conditions (*n* = 4117); however, long-term treatment had more unique genes (*n* = 1518) (Supplementary Figure S6A). We performed gene set enrichment analysis (GSEA) for the genes uniquely accessible in long-term TGFβ conditions and found a high enrichment in gene sets regulated by H3K4me3/H3K27me3 chromatin bivalency and neuronal development (Supplementary Figure S6B). Given the enrichment of CTCF motifs in genes related to histone bivalency, we assessed the global levels of relevant histone modifications [82]. Concomitant with CTCF repression patterns, we also observe increases in H3K4me3 and H3K27me3 in TGFβ-treated conditions (Supplementary Figure S6C).

### 3.6. CTCF Knockdown Enhances Epithelial Traits

**Figure 5:**
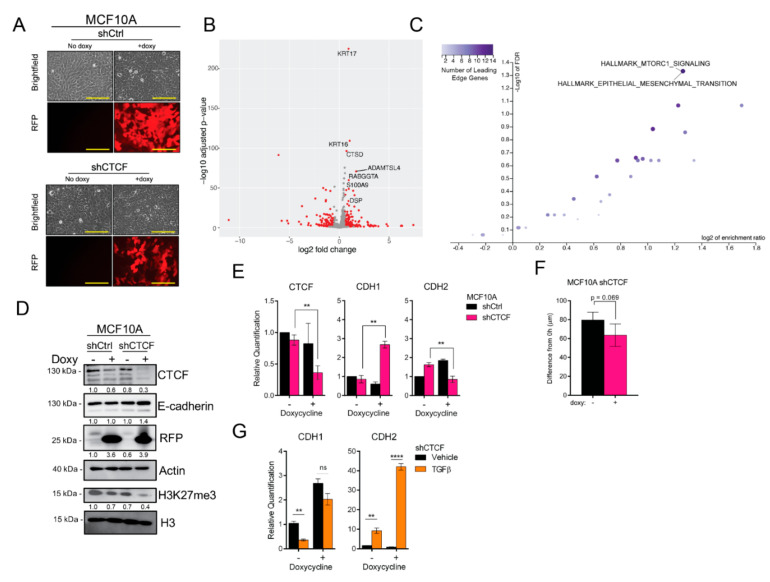
CTCF suppression enhances epithelial traits. (**A**) Representative images of TRIPZ-non-targeting control (shCtrl) and TRIPZ-shRNA against *CTCF* (shCTCF) MCF10A cells with and without 2 days 3.0 µg/mL doxycycline (doxy) treatment. Scale bars = 200 µm. (**B**) Volcano plot indicating transcripts with statistically (FDR < 0.05) and biologically significant fold change (FC > 1.5). (**C**) Gene set enrichment analysis of genes downregulated by CTCF knockdown with hallmark data sets (MSigDB; FDR < 0.05). (**D**) Western blot for indicated proteins in shCtrl- and shCTCF-expressing cells. (**E**) Gene expression measured by qPCR (normalized to *ACTB*) for indicated genes in shCtrl and shCTCF MCF10A cell lines with and without doxycycline (*n* = 3). Statistical significance was tested using a two-tailed Student’s *t*-test. (**F**) Scratch assay for shCTCF-cells with and without doxycycline, showing change in average gap length after 10 h. Error bars indicate s.e.m., (*n* = 4). (**G**) Gene expression measured by qPCR (normalized to *ACTB*) for shCTCF-expressing cells with or without 2d TGFβ treatment (*n* = 3). Statistical significance was tested using a two-tailed Student’s *t*-test. ** *p* ≤ 0.01, **** *p* ≤ 0.0001.

Given our results that show an enrichment for *bona fide* CTCF binding sites within regions with EMT-altered accessibility, we next investigated the effect of CTCF loss-of-expression on EMT. In order to do so, we generated cell lines with a doxycycline-inducible RFP plus shRNA targeting *CTCF.* Given the suppression of CTCF following TGFβ treatment and in mesenchymal breast cancer cell lines, we expected the knockdown to induce mesenchymal traits. However, CTCF knockdown did not change the cellular morphology in comparison to vehicle-treated cells or the doxy-treated non-targeting control (shCtrl) (Figure 5A). Indeed, to our surprise, analysis of gene expression differences by RNA-seq reveals that CTCF knockdown increases the expression of epithelium-associated genes, *KRT16, KRT17, S100A9,* and *DSP* (Figure 5B). Furthermore, the hallmark EMT gene set was enriched in the set of genes downregulated by CTCF knockdown (FDR < 0.05; Figure 5C). We confirmed these effects, noting an increase in E-cadherin protein (Figure 5D) and *CDH1* mRNA (Figure 5E). Concordant with CTCF’s putative involvement in bivalent H3K27me3/H3K4me3 marked genes (Supplementary Figure S6B), the level of H3K27me3 decreases with CTCF knockdown (Figure 5D). To ascertain whether this change in gene expression had a functional effect, we performed migration assays. Consistent with a heightened epithelial state, cells in which CTCF is reduced trended toward a diminished migratory capacity (Figure 5F).

The results of our loss-of function experiments led us to postulate that the loss of CTCF slows the induction of EMT and may serve as a protector of the epithelial phenotype. To investigate this, we induced CTCF knockdown followed by 2 days of TGFβ treatment and assessed changes in *CDH1* and *CDH2* expression. Cells in which CTCF was knocked down failed to significantly suppress *CDH1* expression in the presence of TGFβ, as did control cells (Figure 5G). Despite this, TGFβ treatment induced a greater increase in *CDH2* expression in the CTCF knockdown cells than in control cells (Figure 5G), Thus, the loss of CTCF expression shields *CDH1* from TGFβ-induced reduction, while enhancing the activating effect on *CDH2*, potentially facilitating entrance into a state of partial EMT.

### 3.7. CTCF Overexpression Induces Mesenchymal Traits

**Figure 6:**
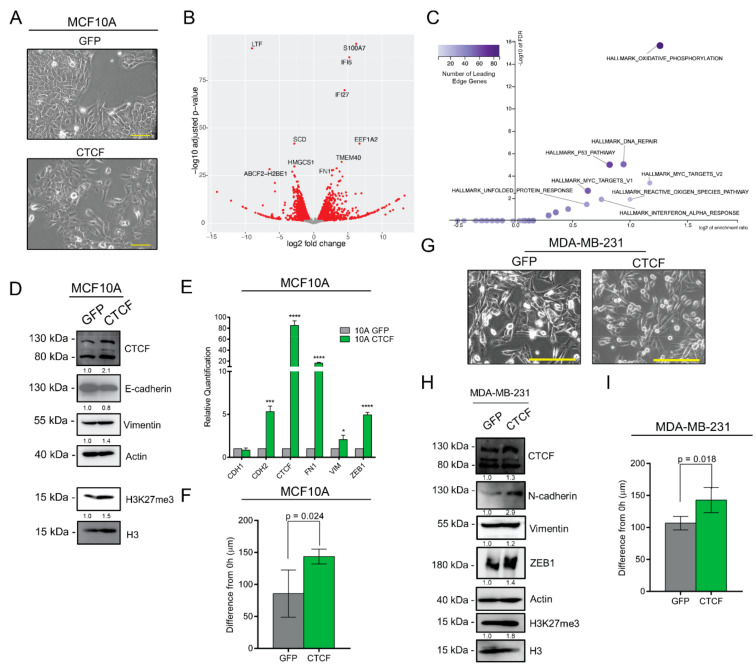
CTCF overexpression enhances mesenchymal gene expression. (**A**) Representative images of pHAGE-GFP (GFP) and pHAGE-CTCF-expressing MCF10A cells. Scale bars = 100 µm. (**B**) Volcano plot indicating transcripts with statistically (FDR < 0.05) and biologically significant fold change (FC > 1.5). (**C**) Gene set enrichment analysis of genes upregulated by CTCF knockdown with hallmark data sets (MSigDB; FDR < 0.05). (**D**) Western blots for indicated proteins in GFP and CTCF-expressing MCF10A cells. (**E**) Gene expression measured by qPCR (normalized to *ACTB*) for indicated genes in GFP and CTCF-expressing cells (*n* = 3). Statistical significance was tested using a two-tailed Student’s *t*-test. * *p* ≤ 0.05, *** *p* ≤ 0.001, **** *p* ≤ 0.0001 (**F**) Scratch assay for GFP- and CTCF-expressing cells. The change in average gap length after 22 h is reported. Error bars indicate s.e.m., (*n* = 4). (**G**) Representative images of pHAGE-GFP (GFP) and pHAGE-CTCF-expressing MDA-MB-231 cells. Scale bars = 200 µm. (**H**) Western blots for indicated proteins in GFP and CTCF-expressing MDA-MB-231 cells. (**I**) Scratch assay for GFP- and CTCF-expressing cells. The change in average gap length after 6 h is reported. Error bars indicate s.e.m., (*n* = 4).

We next determined the implications of CTCF upregulation on EMT. In order to do so, we generated CTCF and GFP over-expressing cells. In concordance with our knockdown data, CTCF overexpressing MCF10A cells appear modestly more spindle-like (Figure 6A). Gene expression analysis by RNA-seq shows an upregulation of *FN1* (Figure 6B) yet fails to link to the hallmark EMT gene set (Figure 6C). Nevertheless, western blot and qPCR analyses reveal the CTCF-induced suppression of E-cadherin and upregulation of vimentin protein expression, as well as an increased level of H3K27me3 (Figure 6D) and induction of *CDH2*, *FN1*, *VIM*, and *ZEB1* at the mRNA level (Figure 6E). Confirming a functional impact, CTCF-overexpressing MCF10A cells showed a greater migratory capacity than control cells (Figure 6F). To determine if increasing the CTCF expression elicits similar changes in a breast cancer cell line, we generated MDA-MB-231 cells overexpressing either GFP or CTCF (Figure 6G). Similar to the effect on MCF10A cells, CTCF increases the expression of N-cadherin and ZEB1 (Figure 6H) and enhances the cells’ migratory capacity (Figure 6I). To determine the extent to which the knockdown or overexpression of CTCF affects genes nearby to CTCF binding motifs within accessible chromatin regions, we re-analyzed the RNA-seq data and calculated the enrichment for differentially expressed genes within this set of genes. As expected, there was a highly significant overlap between differentially expressed genes, either from CTCF overexpression (Supplementary Figure S7A,B) or CTCF knockdown (Supplementary Figure S7C,D) and genes nearby to CTCF binding motifs within accessible chromatin from untreated cells (Supplementary Figure S7A,C) or from cells treated with TGFβ for 10 days (Supplementary Figure S7B,D).

In conclusion, we demonstrate that TGFβ-induced EMT is accompanied by global chromatin relaxation and that TGFβ-withdrawal resolves most but not all alterations in chromatin accessibility. We show that the enrichment of CTCF binding motifs is highly dynamic across EMT/MET. Lastly, we demonstrate that CTCF expression is implicated in epithelial–mesenchymal cell plasticity through the modulation of EMT-related gene expression.

## 4. Discussion

EMT and its reversal, MET, are important for normal physiological processes, such as development and wound healing. Together, these processes are hypothesized to endow cancer cells with metastatic abilities. The transcriptomic determination of the EMT status has linked EMT to poor prognostic characteristics, such as claudin-low breast cancer [43], mesenchymal glioblastoma [83], rapamycin resistance in breast cancer [84], radio resistance in prostate cancer [85], and immune system suppression [86]. Additionally, cells undergoing EMT progress into and through partial states that can assume various morphologies, gene expression, and E-cadherin profiles, and exhibit greater pathogenic properties [68,87]. An examination of solid tumor models has shown that cells within an intermediate-mesenchymal state are more spheroidogenic and resistant to anoikis [40]. Further, cells that express both KRT14 and vimentin disproportionately contribute to metastasis [36]. Despite this importance, the factors that mediate partial- and full-EMT states are not well-characterized. In this study, we have characterized the chromatin accessibly alterations and gene expression output that occur during distinct states of reversible TGFβ-induced EMT.

Herein, we show EMT and MET progress through stage-wise gene expression changes. Many gene expression alterations occur within 48 h of TGFβ treatment. TGFβ treatment rapidly induces Slug (*SNAI2*) mRNA and protein overexpression, which decreases following additional TGFβ treatment and withdrawal (Figure 2D,E). These results suggest that Slug is involved in early EMT induction. *ZEB1* mRNA and protein increase after extended TGFβ treatment, and the *ZEB1*-3′ UTR is quickly suppressed following TGFβ withdrawal (Supplementary Figure S1). These data are dissimilar to those recently reported by Jia et al., who found that the ZEB1 3′UTR reporter remains expressed despite prolonged TGFβ exposure and withdrawal [88]. However, our results corroborate the findings by Ye et al., who report that partial-EMT states are coordinated by Slug, whereas ZEB1 and SNAI1 promote a complete mesenchymal phenotype [89], and data from Addison et al., who show that Slug and ZEB1 EMT-TFs both contribute to E-cadherin suppression but fail to up-regulate the expression of each other [90]. Our data reveal distinctions between short- and long-term TGFβ treatments through the sequential activation of EMT-TFs.

We also characterized EMT/MET states in terms of surface-localized protein markers. To our surprise, we discovered that E-cadherin is lost from the membrane within 2 days of TGFβ treatment, despite robust total E-cadherin and *CDH1* expression. Further TGFβ treatment (4 and 10 days) yields a further suppression of surface E-cadherin. Interestingly, TGFβ withdrawal following long-term treatment stimulates a return of surface E-cadherin^hi^ populations (Figure 1D) despite suppressed *CDH1* and total E-cadherin expression (Figure 2D,E). Such a recovery of surface E-cadherin suggests the presence of a cytoplasmic store capable of returning to the membrane without transcriptional upregulation. This finding is in concert with studies on E-cadherin recycling [91], including reports of the interaction between E-cadherin and late recycling endosome vesicles via Rab5 and Rab11 [73] and recent studies in MCF10A cells demonstrating that extended TGFβ treatment is necessary to induce the loss of membrane-localized E-cadherin [68]. Importantly, we show that TGFβ withdrawal generates a hybrid E-cadherin^hi^/CD44^hi^/CD24^lo^ population, which, given the controversial role of membrane-bound E-cadherin in metastasis [92], may have functional implications in breast cancer progression.

Through an interrogation of Tn5-accessible chromatin, we identify that TGFβ treatment leads to wide-spread alterations in chromatin accessibility. Extensive chromatin alterations occur within 2 days of TGFβ treatment—revealing 22,354 more ATAC peaks and sheltering 9678 peaks. Motif enrichment analyses reveal that TGFβ increases the enrichment for AP-1 and SMAD family binding motifs, transcription factors which have been reported to regulate EMT within accessible regions [35,36,65,93,94]. Withdrawal from TGFβ induces global chromatin constriction, lowering motif enrichment and nearly resolving chromatin to the untreated state. These results demonstrate that chromatin is highly responsive to TGFβ treatment.

Whereas most transcription factor motifs either uniformly increase or decrease following TGFβ treatment, the enrichment of CTCF and BORIS binding elements declines during intermediate states. This suggests a period of chromatin re-shuffling, preparing chromatin for configurations that would promote plasticity through phenotypic states. Notably, Pastushenko et al. also identified CTCF as a factor that is important to EMT states [36]. In their investigation, they isolated populations of epithelial (Epcam^+^), intermediate (CD51^−^/CD61^−^), and mesenchymal (CD106^+^/CD51^+^/CD61^+^) squamous cell carcinoma cells and subjected them to ATAC-seq [36]. Motif enrichment analyses reveal that CTCF motifs were highly enriched in cells exhibiting epithelial or mesenchymal features, but not intermediate states.

In our study, we demonstrate that CTCF, a master chromatin organizer, is dynamically expressed in reversible EMT. EMT-inducing signals reduce CTCF expression, whereas MET restores CTCF expression. We also identified that EMT-related genes (*CDH1*, *CDH2*, *SNAI2,* and *ZEB1*) contain nearby CTCF binding sites—many of which demonstrate dynamic peak profiles during the course of reversible EMT. Recently, multiple studies have implicated CTCF binding key features of cancer biology, including apoptosis. For example, Kaiser et al. probe transcription factor binding sites across 11 tumor types and identify that CTCF binding sites carried high mutational loads [95]. They posit that the mutations alter chromatin landscapes, replication timing, and DNA fidelity within tumors. Further, DNA methylation is well-characterized to affect CTCF binding, as methylation at CTCF motifs can drive the overexpression of oncogenes such as *PDGFRA* [96]. Studies focusing on CTCF protein structure and DNA binding kinetics have identified an alternatively spliced isoform, CTCF-s, which lacks the zinc finger domains that associate with cohesin complex proteins [80]. CTCF-s competes with canonical CTCF for binding sites, consequently imposing variations in chromatin looping and gene neighborhoods [80]. Indeed, we observe a consistent loss of the CTCF-s isoform in cells with mesenchymal features (Figure 4E–G and Supplementary Figure S5B,C).

Through our investigation of the functional consequences of CTCF gain- and loss-of-function on EMT, we demonstrate that CTCF knockdown enhances the epithelial phenotype and suppresses mesenchymal markers. These findings are concordant with Zhao et al., who found that CTCF knockdown suppresses invasion and migration, proliferation, and ovarian cancer metastasis [97]. Unexpectedly, we found that CTCF suppression also decreases H3K27me3, a histone modification tightly linked to EMT-induced gene bivalency [46]. Conversely, the constitutive overexpression of CTCF induces a more mesenchymal phenotype, including the upregulation of mesenchymal markers and increased migratory speed, as well as increased global levels of H3K27me3. Given the findings of Zhang et al., who determined that CTCF overexpression is linked to poor prognoses in patients with hepatocellular carcinoma [98], these findings could have considerable relevance to breast cancer. Though beyond the scope of this study, it would be noteworthy to observe how TGFβ-induced CTCF suppression affects 3D chromatin structure and topological domains.

## 5. Conclusions

In conclusion, our study demonstrates that mammary epithelial cells proceed through EMT and MET via progressive and partially reversible chromatin accessibility alterations. We reveal that EMT is marked by global chromatin loosening and that MET is marked by chromatin constriction. We show that a subset of differentially accessible ATAC peaks are enriched for CTCF binding motifs and we demonstrate that CTCF overexpression can promote an EMT-like state in mammary epithelial cells. Collectively, our findings indicate that the activation of EMT and MET dramatically reconfigures chromatin organization, and that CTCF is a key modulator of epithelial–mesenchymal plasticity through the modulation of epithelial and mesenchymal gene expression.

## Data Availability

The ATAC and RNA sequencing data generated in this work have been deposited in NCBI’s Gene Expression Omnibus and are accessible though GEO series accession number GSE145851. ATAC-seq files are also accessible through the UCSC genome browser [https://genome.ucsc.edu/s/kelsey_johnson1/Reversible%20EMT%20ATAC%2Dseq%20peaks].

## Supplementary Materials

The following supporting information can be downloaded at: https://www.mdpi.com/article/10.3390/cancers14010209/s1, Figure S1: TGFβ treatment suppresses CDH1 promoter reporter activity and increases ZEB1 3′UTR reporter activity. Figure S2: Dynamics of TGFβ-induced and TGFβ-withdrawn chromatin accessibility. Figure S3: EMT, mammary basal cell, and stemness gene sets are enriched in TGFβ-induced and persistent peaks. Figure S4: GIGGLE similarity scores of probable chromatin accessibility regulators at various stages of the EMT-MET spectrum in breast-specific cell lines. Figure S5: EMT suppresses total CTCF protein levels. Figure S6: Genes near accessible CTCF motifs are enriched for bivalent, neuronal, and signal transduction genes. Figure S7: Change in gene expression upon CTCF manipulation in MCF10A cells overlap with genes nearby to accessible chromatin with CTCF motifs.
