# Supplementary material for: CTCF Expression and Dynamic Motif Accessibility Modulates Epithelial–Mesenchymal Gene Expression
Source: Cancers (Basel). 2022 Jan 1;14(1):209. doi: 10.3390/cancers14010209 (PMC8750563; doi:10.3390/cancers14010209)
Supplement: Supplementary file 1 [file cancers-14-00209-s001.zip › cancers-1279773-supplementary.pdf]

cancers

MDPI

Article

CTCF Expression and Dynamic Motif Accessibility Modulates Epithelial–Mesenchymal Gene Expression

Kelsey S. Johnson [1], Shaimaa Hussein [2], Priyanka Chakraborty [3], Arvind Muruganantham [1], Sheridan Mikhail [1], Giovanny Gonzalez [1], Shuxuan Song [1], Mohit Kumar Jolly [3], Michael J. Toneff [4], Mary Lauren Benton [5], Yin C. Lin [2] and Joseph H. Taube [1,6,*]

[1] Department of Biology, Baylor University, Waco, TX 76706, USA; kelsey_johnson1@baylor.edu (K.S.J.); arvind_murugananthal@baylor.edu (A.M.); sheridan_mikhail1@baylor.edu (S.M.); giovanny_gonzalez1@alumni.baylor.edu (G.G.); shuxuan_song1@baylor.edu (S.S.)
[2] Baylor Institute for Immunology Research, Baylor Scott & White, Dallas, 75246 TX, USA; Shaimaa.zaki@gmail.com (S.H.); linchunyin@gmail.com (Y.C.L.)
[3] Centre for BioSystems Science and Engineering, Indian Institute of Science, Bangalore 560012, India; priyanka08993@gmail.com (P.C.); mkjolly@iisc.ac.in (M.K.J.)
[4] Department of Biology, Widener University, Chester, PA 19013, USA; mtoneff@widener.edu
[5] Department of Computer Science, Baylor University, Waco TX, 76706, USA; marylauren_benton@baylor.edu
[6] Dan L. Duncan Cancer Center, Houston, TX, USA
* Correspondence: Joseph_Taube@baylor.edu

Supplementary

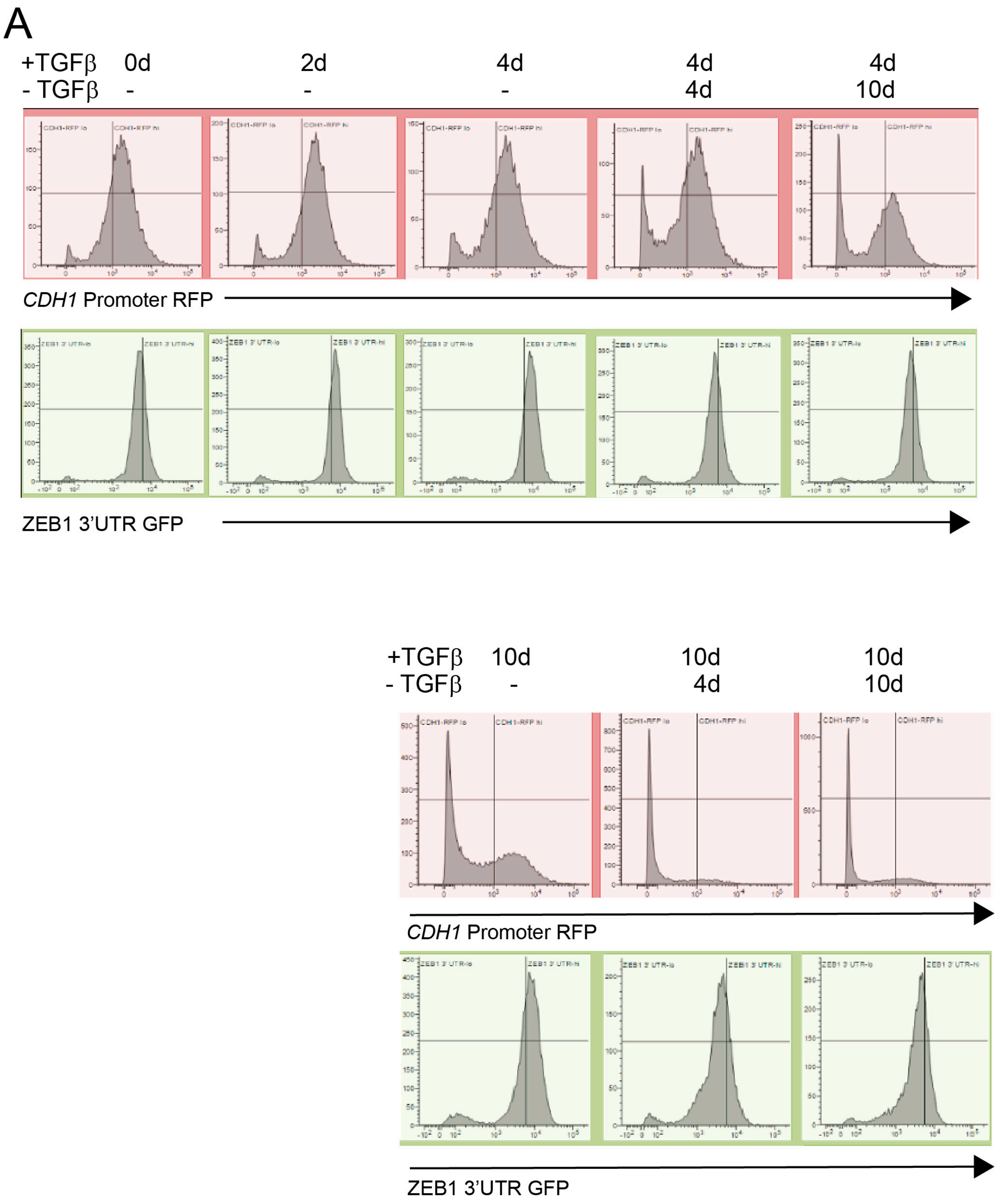

Figure S1. TGFβ treatment suppresses *CDH1* promoter reporter activity and increases ZEB1 3'UTR reporter activity. **(A)** FACS profiling of *ZEB1* 3' UTR reporter and *CDH1* promoter reporter activity. FACS quantification for Z1-GFP reporter (green panel), *CDH1*-RFP reporter (pink panel).

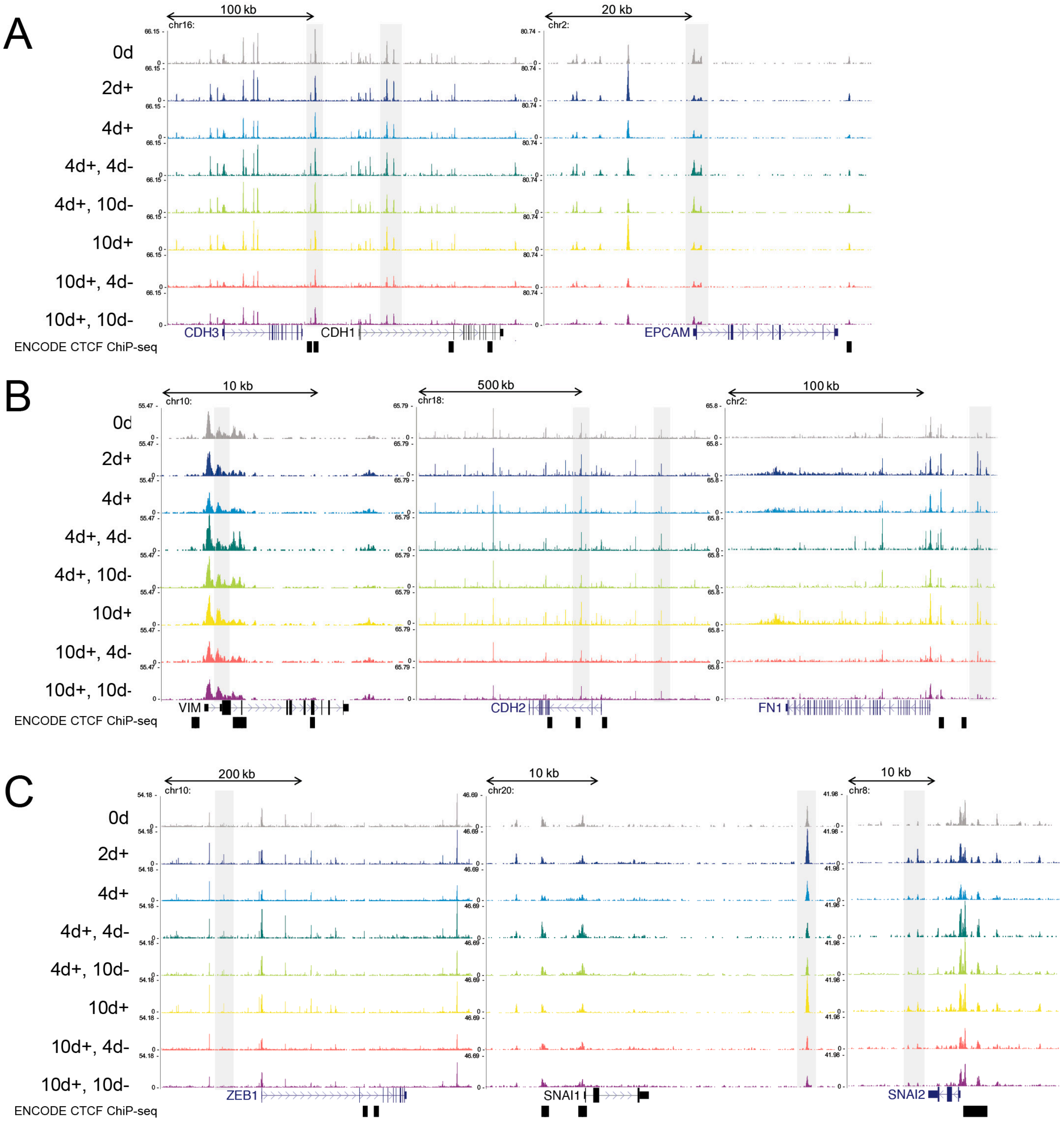

Figure S2. Dynamics of TGFβ-induced and TGFβ-withdrawn chromatin accessibility. ATAC-seq peak profiles of select **(A)** epithelial (*CDH3*, *CDH1*, and *EPCAM*), **(B)** mesenchymal (*VIM*, *CDH2*, and *FN1*), and **(C)** EMT-TF (*ZEB1*, *SNAI1*, and *SNAI2*) genes at indicated short-term (top) and long-term (bottom) TGFβ treatment models ($n = 2$). Black rectangles below genes refer to ENCODE CTCF ChIP-seq peaks in human mammary epithelial cells (HMEC).

Table 1

	Hallmark Gene Sets
1	UV Response DN
2	Estrogen Response Early
3	Epithelial mesenchymal transition
4	TNFα Signaling via NFκB
5	Apoptosis
6	IL2 STAT5 Signaling
7	Hypoxia
8	Mitotic Spindle
9	TGFβ Signaling
10	Androgen Response
11	Inflammatory Response
12	Hedgehog Signaling
13	Apical Junction
14	p53 Pathway
15	Xenobiotic Metabolism
16	KRas Signaling Up

Table 2

	Breast and Mammary-Specific Gene Sets	Study
A	Mammary Stem Cell Up	Lim et al., 2010
B	Luminal vs Mesenchymal Down	Charafe et al., 2006
C	Luminal vs Basal Down	Charafe et al., 2006
D	Pubertal Breast 4/5wk Up	McBryan et al., 2007
E	Basal vs Luminal	Farmer et al., 2005
F	Luminal B Down	Smid et al., 2008
G	Pubertal Breast 3/4wk Up	McBryan et al., 2007
H	Basal Up	Smid et al., 2008
I	Invasive Breast Cancer Down	Poola et al., 2005
J	Breast Cancer Relapse in Bone Down	Smid et al., 2008
K	Ductal Invasive Up	Schuetz et al., 2006
O	Basal Down	Smid et al., 2008
L	Medullary vs Ductal Breast Cancer Down	Bertucci et al., 2006
M	Luminal Mature Down	Lim et al., 2010
N	Ductal Invasive Down	Schuetz et al., 2006
P	ESR1 Laser Up	Yang et al., 2006
Q	Copy Number Down	Climent et al., 2007
R	Luminal vs Basal Up	Charafe et al., 2006

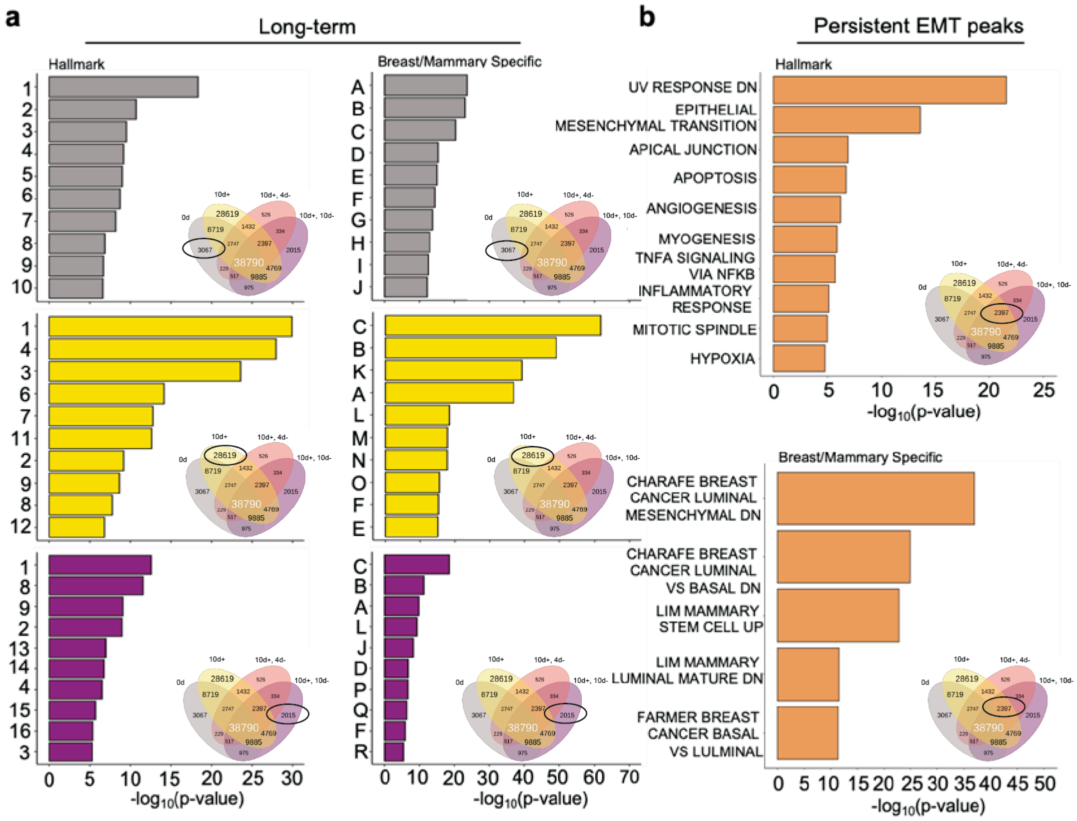

Figure S3. EMT, mammary basal cell, and stemness gene sets are enriched in TGFβ-induced and persistent peaks. (Table 1) Number key for GSEA MSigDB hits for Hallmark gene sets. (**Table 2)** Letter key for GSEA MSigDB hits for breast and mammary-specific gene sets. **(A)** GSEA MSigDB hits (based on number key in Table 1) for ATAC peaks in Hallmarks gene sets (left) and hits (based on letter key in Table 2) for breast and mammary-specific gene sets (right) in long-term TGFβ-induced and –withdrawn conditions. Gray = 0d; yellow = 10d + TGFβ; purple = 10d + TGFβ, 10d −TGFβ. **(B)** GSEA MSigDB hits for genes annotated to ATAC peaks with persistent chromatin alterations following TGFβ treatment and withdrawal in Hallmarks gene sets (top) and breast and mammary-specific gene sets (bottom).

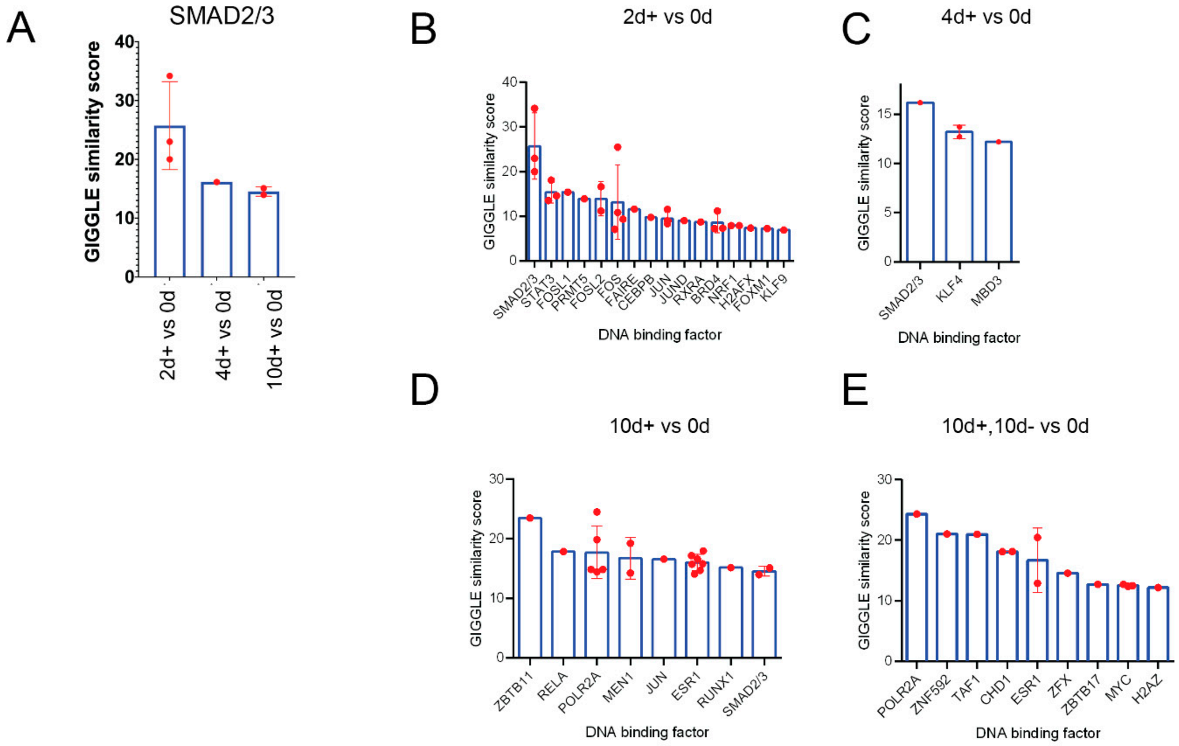

Figure S4. GIGGLE similarity scores of probable chromatin accessibility regulators at various stages of the EMT-MET spectrum in breast-specific cell lines. Peaks enriched at the indicated timepoints vs untreated cells were compared to genome interval files. (**A**) SMAD2/3-associated genome interval files show a diminished score as EMT progresses. (**B–E**) All scoring genome interval files for the indicated timepoint comparisons are shown. Data points refer to independent genome interval files queried by GIGGLE.

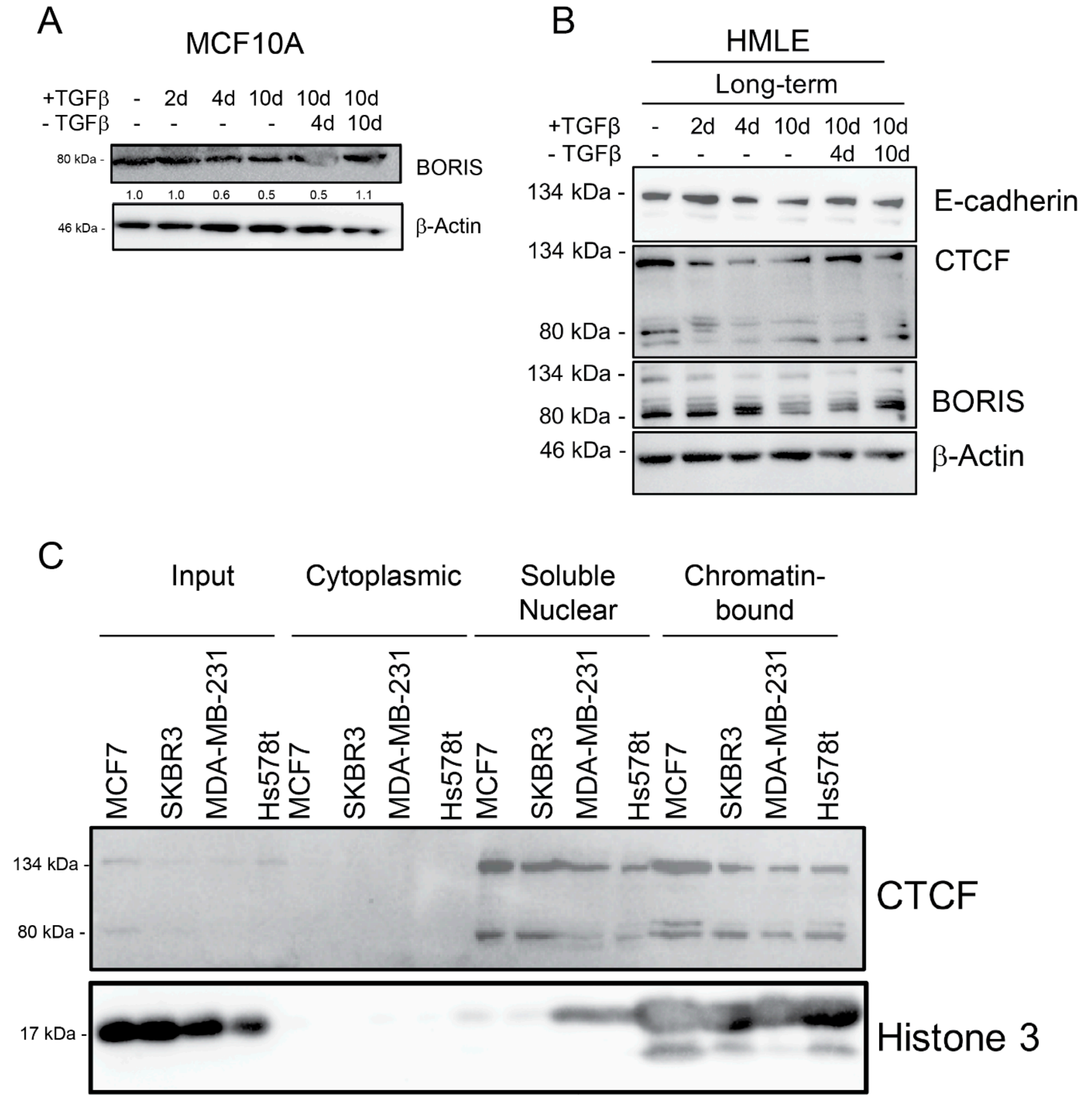

Figure S5. EMT suppresses total CTCF protein levels. **(A)** Western blot for BORIS in treated MCF10A cells. **(B)** Western blot for E-cadherin, CTCF, and BORIS in HMLE cell lines treated with (or withdrawn from) 5 ng/mL TGFβ at the indicated time. **(C)** Western blot for CTCF localization in specified cell fractions in the specified breast cancer cell lines.

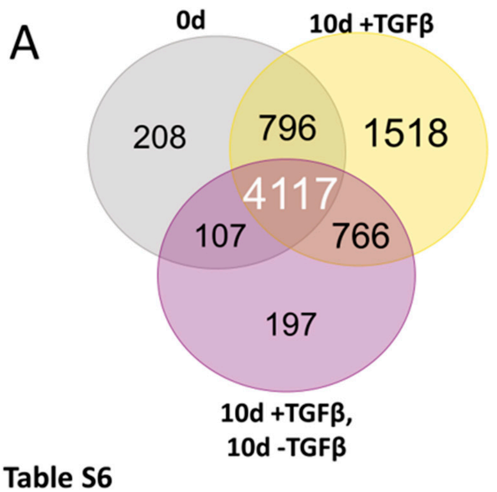

Table S6

Number Key for all GSEA msigDB hits

	Gene Set
1	CCANNAGRKGGC UNKNOWN
2	MEISSNER BRAIN HCP WITH H3K4ME3 AND H3K27ME3
3	CAGGTG V$E12 Q6
4	GO NEURON PART
5	GO REGULATION OF INTRACELLULAR SIGNAL TRANSDUCTION
6	GO NEUROGENESIS
7	ONDER CDH1 TARGETS 2 DN
8	MEISSNER NPC HCP WITH H3K4ME2
9	GO POSITIVE REGULATION OF CELL COMMUNICATION
10	KOINUMA TARGETS OF SMAD2 OR SMAD3
11	BENPORATH ES WITH H3K27ME3
12	GO CELL DEVELOPMENT
13	GO CELL PROJECTION
14	GO REGULATION OF CELL PROJECTION ORGANIZATION
15	PEREZ TP53 TARGETS
16	GO RESPONSE TO OXYGEN CONTAINING COMPOUND
17	chr1q32

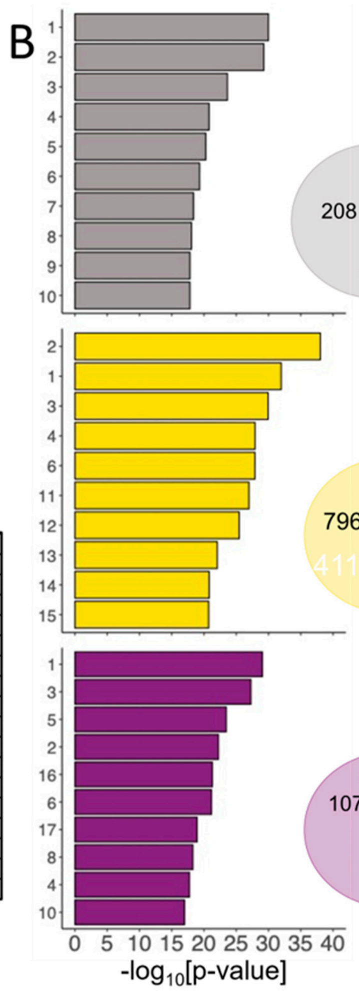

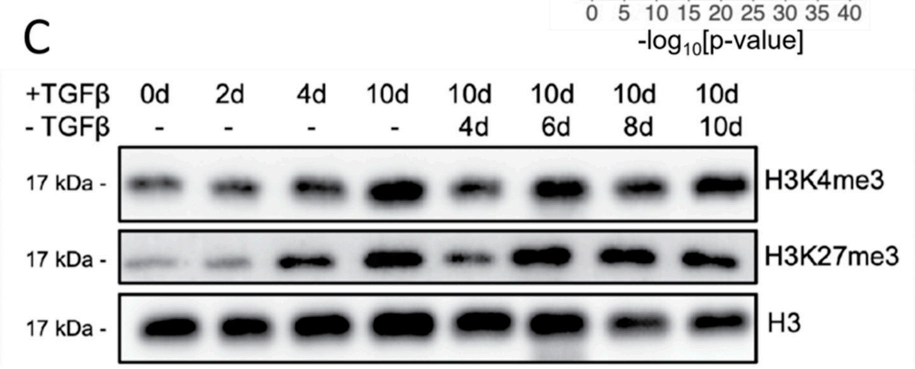

Figure S6. Genes near accessible CTCF motifs are enriched for bivalent, neuronal, and signal transduction genes. **(A)** Venn diagram representing the number of genes adjacent to accessible CTCF motifs at the indicated timepoints. **(Table S6)** GSEA MSigDB gene sets determined to be highly-enriched in accessible CTCF motifs. **(B)** GSEA MSigDB enrichment (based on number key in Table S6) for top-10 enriched gene sets by condition circled in venn diagram to the right. **(C)** Western blot for histone modification changes in treated MCF10A cells.

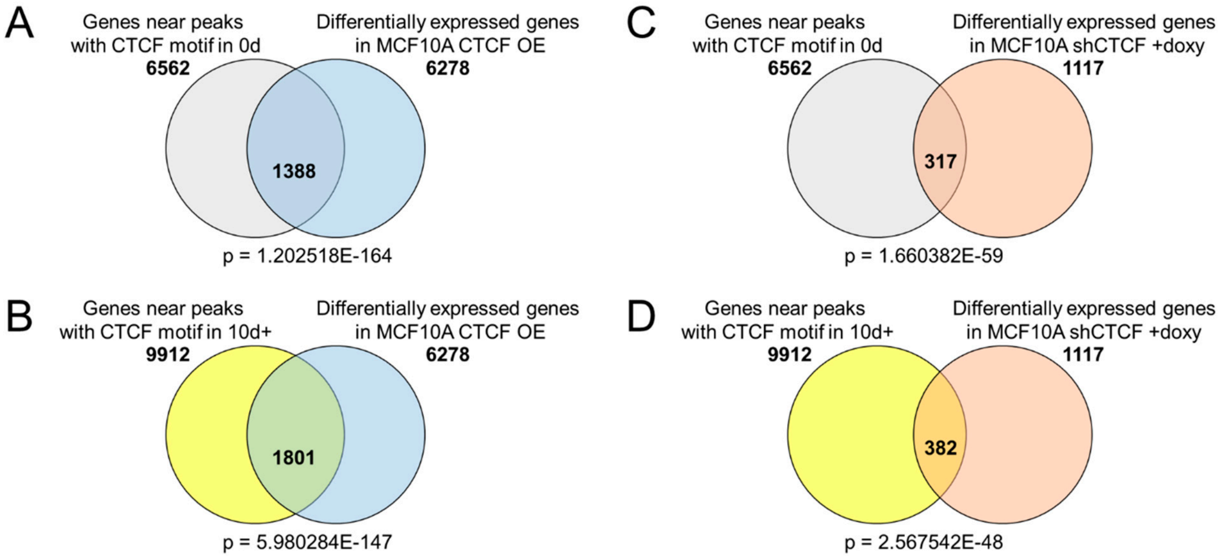

Figure S7. Change in gene expression upon CTCF manipulation in MCF10A cells overlap with genes nearby to accessible chromatin with CTCF motifs. Overlap between genes differentially expressed in CTCF overexpression MCF10A cells (blue-A,B) or in CTCF knockdown MCF10A cells (red-C,D) and the set of genes nearest to ATAC-seq peaks containing a CTCF motif from either the untreated MCF10A cells (gray-A,C) or MCF10A cells treated with TGFβ for 10 days (yellow-B,D). p-value calculated using a hypergeometric test.
